# FDA-approved phensuximide inhibits RIPK1-dependent immunogenic cell death

**DOI:** 10.1038/s41419-025-07754-2

**Published:** 2025-06-02

**Authors:** Byeong-Ju Kim, Sun Mi Hong, Hyun-Jin Noh, Jihye Kim, Su-Yeon Seon, Jeong-Eun Lee, Da-Hye Jeong, Ju-Mi Park, Sejeong Park, Sanghoon Lee, Jaewoo Kang, Dakeun Lee, Michael J. Morgan, You-Sun Kim

**Affiliations:** 1 Department of Biochemistry, Ajou University School of Medicine, Suwon, 16499 Republic of Korea; 2 Department of Biomedical Sciences, Graduate School of Ajou University, Suwon, 16499 Republic of Korea; 3 AIGEN Sciences, Seoul, Republic of Korea; 4 Department of Computer Science and Engineering, Korea University, Seoul, 02841 Republic of Korea; 5 Department of Pathology, Ajou University School of Medicine, Suwon, 16499 Republic of Korea; 6 Department of Natural Sciences, Northeastern State University, Tahlequah, OK USA

**Keywords:** Necroptosis, Cell death and immune response

## Abstract

Receptor-interacting serine/threonine kinase 1 (RIPK1) is a pivotal protein controlling cell death and inflammation. RIPK1 is an attractive therapeutic target, given that the inhibition of RIPK1 kinase activity has been shown to be effective in animal models of human diseases such as autoimmune and neurodegenerative diseases. Here, we screened a collection of drugs with structural similarity to necrostatin-1 (Nec-1), an inhibitor of RIPK1, to assess their abilities to regulate RIPK1-mediated immunogenic cell death. Through this small-scale screening of drugs from ongoing clinical trials and FDA-approved drugs, we discovered that the drug phensuximide could prevent necroptosis by targeting RIPK1 kinase activity. Importantly, phensuximide, which has already been approved by the FDA for the treatment of epilepsy, effectively prevents the kinase activity of RIPK1 without affecting the NF-κB and MAPK pathways. The potency of phensuximide is that it protects against both LPS- and TNF-induced systemic inflammatory response syndrome (SIRS), which are sepsis models involving RIPK1 kinase activity. Our findings suggest that phensuximide may serve as a promising strategy for targeting RIPK1-mediated diseases.

## Introduction

Receptor-interacting serine/threonine kinase 1 (RIPK1) is a central regulator of survival, cell death, and inflammation in response to numerous stimuli [1–3]. Whereas RIPK1 scaffold activity is involved in the activation of the canonical NF-κB and MAPK pathways, the kinase activity of RIPK1 plays a pivotal role in determining cell fate during RIPK3-mediated necroptosis, an immunogenic form of cell death [4–6]. RIPK1 kinase activity has therefore been implicated in mediating or exacerbating pathological conditions across a spectrum of inflammatory or degenerative diseases, including rheumatoid arthritis, sepsis, inflammatory bowel disease (IBD), ischemia, psoriasis, and multiple sclerosis [7–9]. Notably, inhibition of the RIPK1 kinase has shown significant promise in providing robust and consistent protection against sepsis, mitigating sepsis-induced hypothermia and preventing its lethality [10]. These findings suggest the importance of RIPK1 kinase activity in perpetuating immune hyperreactivity postinfection.

RIPK1 is considered a promising therapeutic target, and many RIPK1 inhibitors have been developed to date with the hope that they could be useful in the clinic [11–13]. Necrostatin-1 (Nec-1), the first RIPK1 kinase inhibitor, binds to an allosteric pocket, hindering the conformational rearrangement of both the N-lobe and C-lobe, along with the backside of the kinase domain [7, 14]. A stable version of Nec-1 (Nec-1s) has been widely used in research to investigate the mechanism of necroptosis and disease models and to understand the role of RIPK1 kinase activity in these settings [14, 15]. However, this compound is limited by its very short half-life in vivo and other limitations, along with its poor metabolic stability, which limits its clinical relevance [16, 17]. Others have been designed targeting the allosteric pocket adjacent to the ATP-binding site of RIPK1, which maintains an unusual DLG-out/Glu-out inactive conformation that regulates the kinase activity of RIPK1 [18, 19]. Clinical trials of RIPK1 inhibitors in patients are still in their infancy, and to date, a few chemotypes of RIPK1 inhibitors with appropriate in vivo properties have been developed. The first compounds investigated for RIPK1 inhibition were necrostatin-1 derivatives, identified through structure-activity relationship (SAR) studies. Additionally, other inhibitors have been developed based on scaffold compounds such as PK6 or natural products [13]. Many have been isolated from GlaxoSmithKline or from other kinase inhibitor libraries. A few small-molecule inhibitors of RIPK1, such as GSK’2982772 and the Denali Therapeutics/Sanofi compounds DNL747, DNL758 and DNL788, exhibit high selectivity and have progressed to phase I/II clinical trials in humans (source: ClinicalTrials.gov) [20, 21]. Additionally, Genentech’s compound GDC-8264 has entered clinical trials for Graft Versus Host disease and Rigel/Eli Lilly R552 and Sironax’s SIR2446 are now in Phase II trials for the treatment of inflammatory conditions. However, no drugs specifically designed as RIPK1 inhibitors have yet been approved for clinical use.

With no approved drugs currently on the market, some labs have turned to drug repositioning/drug repurposing for clinical compounds that are already approved for an indication other than the one for which they were initially marketed [22]. Drug repositioning makes the initial phases of drug development considerably faster and cheaper, and increases the chances of introducing it on the market, in large part because toxicity studies have already been performed and the drug has proven safe [23]. Because of similarity between kinase domains, several compounds that were designed for other kinases have shown to inhibit RIPK1. For instance, many inhibitors of Aurora Kinases (AurK), Phosphoenolpyruvate Carboxykinase (PEPK), and RAF have been shown to have off-target effects that inhibit RIPK1 [13]. Some of these, such as Debrafenib and Vemurafenib, are approved for use in the clinic to treat cancers. Pazopanib and Ponatinib are tyrosine kinase inhibitors (TKI) that inhibit VEGFR and BCR-ABL fusion proteins, respectively, but also inhibit RIPK1 [24]; the former is used in the treatment of soft tissue sarcomas and the latter is used to treat CML and ALL. Phenytoin and Primidone are anti-epileptic medications that have also been shown to inhibit RIPK1 in vitro and in vivo [25, 26].

Approaches used for drug repurposing can be subdivided into computational approaches and experimental approaches [27]. Various computational approaches can be used individually or in combination to systematically analyze different types of large-scale data, such as gene expression, chemical structure, genotype, proteomic data or electronic health records [28]. Among them, molecular docking is a structure-based computational strategy to predict binding site complementarity between a drug and a therapeutic target protein. In this study, we employed a computational screening process to identify potential inhibitors of RIPK1. We screened over 10,000 drug-like molecules based on their structural similarity to Nec-1. By focusing on molecules that share structural characteristics with Nec-1, we analyzed compounds that are likely to engage key functional domains on RIPK1 and inhibit its activity. Through this screening process, we identified the anti-epilepsy drug phensuximide (Phen) as a novel inhibitor of RIPK1 kinase activity. We demonstrated that Phen effectively prevents RIPK1-dependent necroptosis without affecting the NF-κB and MAPK pathways and does not affect apoptotic events that are not reliant on RIPK1 kinase activation. Additionally, Phen mitigated RIPK1-driven inflammation in a murine model of systemic inflammatory response syndrome (SIRS) induced by lipopolysaccharide (LPS) and tumor necrosis factor (TNF). These findings suggest that Phen, which is already approved by the FDA for epilepsy treatment, may represent a novel therapeutic strategy for targeting RIPK1-mediated diseases.

## Results

### Identification of phensuximide as a potential RIPK1 inhibitor

**Fig. 1:**
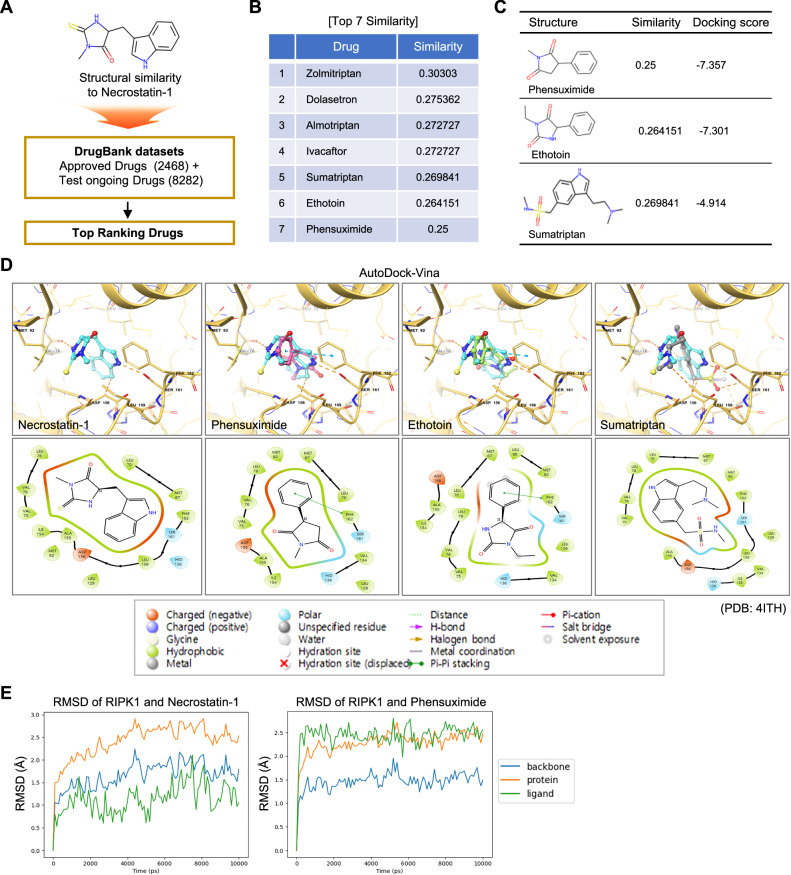
Identification of highly similar drugs to necrostatin-1. **A**, **B** Top-ranking drugs similar to necrostatin-1. Drugs sharing structural similarity were ranked from the DrugBank dataset (A), and the top 7 drugs and their similarity values to Nec-1 are displayed (B). **C** Comparison of docking scores for phensuximide (Phen), ethotoin, and sumatriptan with the RIPK1 kinase domain and their corresponding chemical structures. **D** Molecular docking analysis depicting the binding modes of Nec-1, Phen, ethotoin and sumatriptan within the RIPK1 kinase domain. Key interactions, including hydrogen bonds and π‒π stacking interactions, are highlighted in the upper panel. Amino acids of RIPK1 within 4Å are displayed with ligands in the lower panel, with the properties of the amino acids and their interactions with ligands annotated in the accompanying box. **E** Molecular dynamics (MD) simulation showing the root-mean-square deviations (RMSDs) of the RIPK1 kinase domain and ligands necrostatin-1 (left panel) and phensuximide (right panel).

We screened for compounds structurally similar to Nec-1 to develop potential inhibitors of RIPK1 kinase activity-dependent necroptosis. Leveraging the established drug database DrugBank, which includes 2,468 approved drugs and 8282 experimental drugs, we identified several candidates that share structural similarities with Nec-1 (Fig. 1A, B and Fig. S1A). Following the identification of structurally similar compounds, we analyzed their potential interactions with RIPK1 and compared them with Nec-1 via molecular docking studies. Although several of these compounds had highly similar structures to Nec-1, phensuximide (Phen, trade name:Milontin) and ethotoin had high docking scores and were better than sumatriptan (Fig. 1C). The highest docking score in the computational simulation was obtained for Phen. Belonging to the succinimide class, Phen exhibits anticonvulsant properties by inhibiting the paroxysmal EEG pattern characterized by three cycles per second spike and wave activity, which is associated with episodes of impaired consciousness in petit mal seizures [29, 30]. In a computational simulation using AutoDock Vina, Nec-1 was observed to form hydrogen bonds with Ser161 and Asp156 within the RIPK1 kinase domain, which is consistent with findings in the literature (Fig. 1D). These interactions are critical for stabilizing the kinase in its inactive conformation, thereby inhibiting the activation of necroptosis pathways [18]. Specifically, Ser161 plays a key role in modulating the RIPK1 activation state by influencing the kinase’s conformational dynamics, and the binding of Nec-1 to Ser161 impedes phosphorylation, maintaining RIPK1 in an inactive state. Asp156, located near the ATP-binding site, is instrumental in preserving the structural integrity of this region, and its interaction with Nec-1 alters the geometry of the ATP-binding pocket, obstructing ATP binding and subsequent autophosphorylation, further inhibiting kinase activity. Phen exhibited a binding mode comparable to that of Nec-1. A significant π‒π stacking interaction was identified between the phenyl ring of Phen and the phenylalanine residue at position 162 (Phe162) of RIPK1. This residue critically contributes to the structural stability of the activation loop, regulating the kinase’s conformational equilibrium between its active and inactive states, which suggests that Phen may stabilize RIPK1 in its inactive conformation in a manner similar to Nec-1 (Fig. 1D). Ethotoin, another structurally similar compound, also exhibited a similar binding mode, while sumatriptan was not predicted to significantly bind to RIPK1, despite its structural similarity (Fig. 1D). Molecular dynamics (MD) simulations also confirmed that Phen formed stable interactions with the RIPK1 binding site; however, the root-mean-square deviation (RMSD) for the ligand Phen showed lower fluctuations and a shorter initial equilibration period than Nec-1 did, indicating the potential for a more stable interaction with RIPK1 (Fig. 1E); however, hydrogen bond (H-bond) dynamics simulations indicated that Phen has fewer hydrogen bonds than Nec-1 does. Nec-1 was predicted to form stable H-bonds with Val76, Asp156, and Ser161, whereas Phen was predicted to form an H-bond with Asp156 of RIPK1 (Fig. S1B, C). These results indicate that the stable binding of Phen with RIPK1 likely results from the strong interaction with Phe162 in the DLG motif of RIPK1.

### Phensuximide potently inhibits TNF-induced necroptotic cell death

**Fig. 2:**
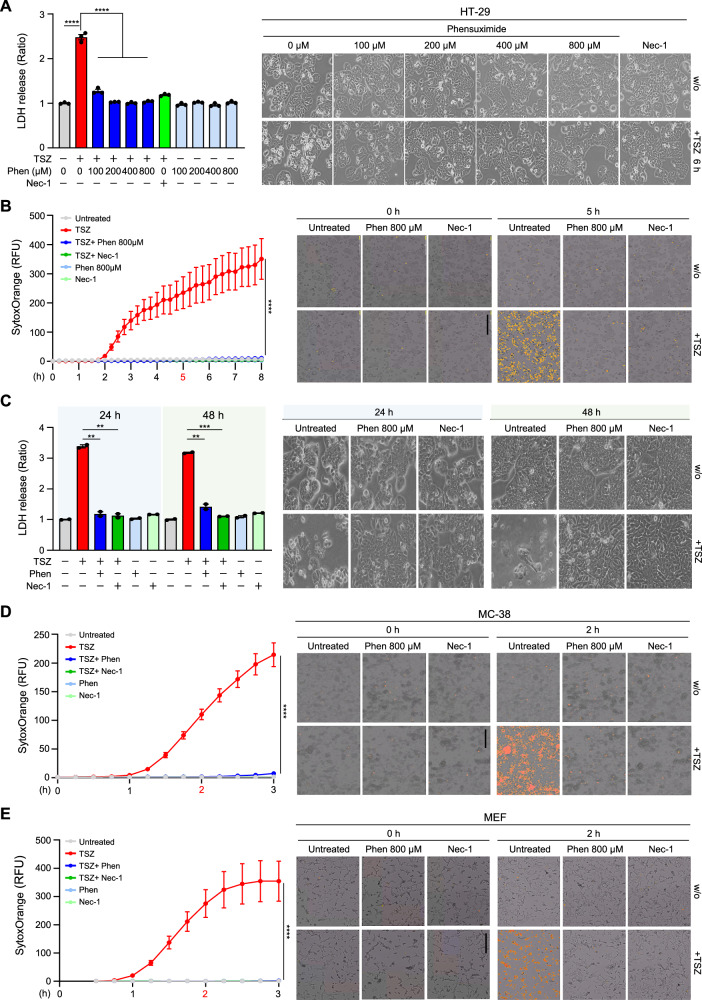
Phensuximide-mediated inhibition of necroptosis is species independent. **A** HT-29 cells were pretreated with the indicated concentrations of Phen or Nec-1 (40 μM) for 1 h and then treated with TNF-α (30 ng/mL) + SMAC (200 nM) + z-VAD (20 μM) for 6 h. Cytotoxicity was determined using the LDH assay (left panel), and images are representative of at least three independent experiments (right panel). **B** HT-29 cells were pretreated with Phen (800 μM) or Nec-1 for 1 h and then treated with TSZ. Cytotoxicity was measured by the fluorescence intensity of Sytox Orange using a Lionheart FX automated microscope (left panel). Representative images of cell death were taken at 5 h (right panel). Scale bar = 400 μm. **C** HT-29 cells were pretreated with Phen or Nec-1 for 1 h and then treated with TSZ for 24 h or 48 h. Cell death was analyzed by the LDH assay (left panel) and representative images of cell death were taken at 24 or 48 h (right panel). **D**, **E** MC-38 cells (D) and MEFs (E) were pretreated with Phen or Nec-1 for 1 h and then treated with TSZ. Cytotoxicity was measured by the fluorescence intensity of Sytox Orange (D and E, left panel). Representative images of cell death were taken at 2 h (D and E, right panel).

**Fig. 3:**
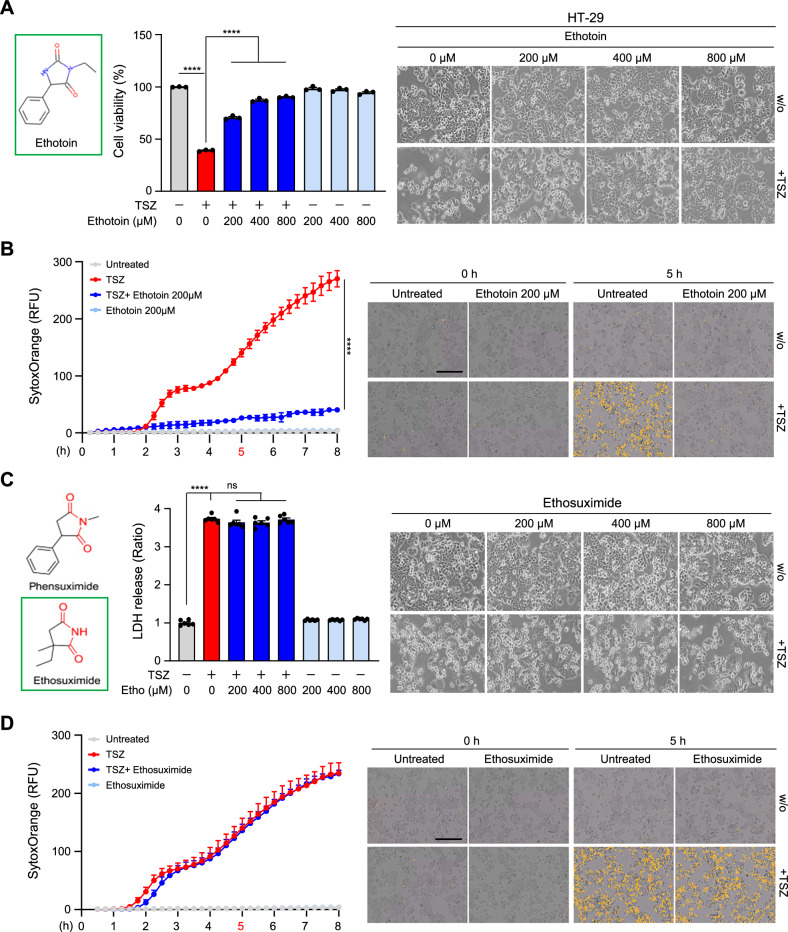
Phensuximide derivatives have no inhibitory effects on necroptosis. **A** Chemical structure of ethotoin (left panel). HT-29 cells were pretreated with the indicated concentrations of ethotoin for 1 h and then treated with TSZ for 5 h. Cell viability was analyzed by the MTT assay (middle panel). Images are representative of at least three independent experiments (right panel). **B** HT-29 cells were pretreated with ethotoin (200 μM) for 1 h and then treated with TSZ. Cytotoxicity was measured by the fluorescence intensity of Sytox Orange (left panel). Representative images of cell death were taken at 5 h (right panel). **C**, **D** Chemical structures of Phen and ethosuximide (C, left panel). HT-29 cells were pretreated with the indicated concentrations of ethosuximide for 1 h and then treated with TSZ. Cytotoxicity was determined by the LDH assay (C, middle panel), representative images of cell death (C, right panel) or the fluorescence intensity of Sytox Orange (D).

We next tested whether Phen actually influences TNF-induced necroptosis. TNF-α plus a Smac mimetic and the pan-caspase inhibitor z-VAD (hereafter referred to as TSZ) is a well-established cocktail that is used to trigger RIPK1-mediated necroptotic cell death. A substantial protective effect of 40 μM Nec-1 on necroptosis during this treatment was observed. We treated HT-29 cells with TSZ in the presence or absence of Phen at concentrations ranging from 25 μM to 200 μM. At concentrations less than 100 μM, Phen inhibited necroptosis (Fig. S2A). Using these concentrations, we evaluated the IC50 of Phen in TSZ-induced cell death (Fig. S2B). When we increased the concentration of Phen to 800 μM, we found that this high concentration did not have any effect on cell cytotoxicity but completely blocked necroptotic cell death (Fig. 2A). This finding was further supported by experiments in which the population of Sytox Orange-positive cells was measured at longer time points (Fig. 2B and Fig. S2C). Notably, Phen protected cells from necroptosis for more than 48 h without any cytotoxicity (Fig. 2C). The inhibitory effect of Phen on TNF-mediated necroptosis was also evident in another human cancer cell line, an ectopic RIPK3-expressing MDA-MB231 cell line, as well as in murine MC-38 cells and MEFs, expanding this observation to multiple cell types and species (Fig. 2D, E and Fig. S2D). The IC50 of Phen was also evaluated in both MC-38 and MEF cells (Fig. S2E, F). We next tested the second-ranked drug ethotoin, a hydantoin derivative that also has anticonvulsant properties and exerts its antiepileptic effect without inducing general depression of the central nervous system [31]. Its mechanism of action is likely very similar to that of phenytoin, which, interestingly, has been proposed to be an inhibitor of necroptosis that may at least partially involve RIPK1 inhibition [26, 32]. At a concentration of 200 μM, similar to phenytoin, ethotoin inhibited TNF-mediated necroptosis, suggesting the validity of our screening strategy for identifying drugs structurally related to Nec-1 with high docking scores to RIPK1 (Fig. 3A, B).

We verified whether other members of the succinimide class have the same effect on necroptosis. Ethosuximide, the most commonly used succinimide, is a valuable agent in pediatric medicine for managing absence seizures [33]. Although Phen had some protective effect at 100 μM, ethosuximide did not have any inhibitory effect on TNF-mediated necroptosis until the concentration reached 800 μM. This high concentration, however, did not result in any cytotoxicity (Fig. 3C, D). Methsuximide, a succinimide-based anticonvulsant similar to Phen, also had no observable effect on TNF-mediated necroptosis, suggesting that the inhibition of necroptosis is specific to Phen (Fig. S3A, B).

### Inhibition of RIPK1 kinase activity by phensuximide treatment

**Fig. 4:**
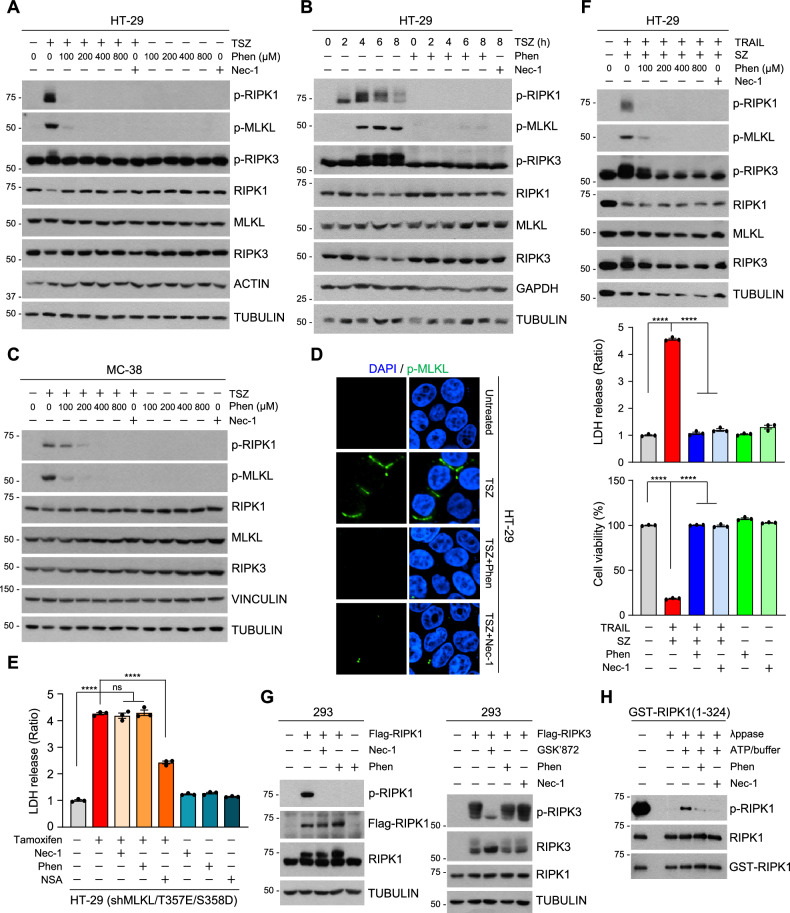
Phensuximide efficiently blocks RIPK1 S166 autophosphorylation. **A** HT-29 cells were pretreated with the indicated concentrations of Phen or Nec-1 for 1 h and then treated with TSZ for 4 h. The cell lysates were analyzed by western blotting. **B** HT-29 cells were pretreated with Phen (200 μM) or Nec-1 for 1 h and then treated with TSZ for the indicated times. **C** MC-38 cells were pretreated with the indicated concentrations of Phen or Nec-1 for 1 h and then treated with TSZ for 2 h. **D** Image of immunocytochemistry staining for p-MLKL in TSZ-, TSZ+Phen-, and TSZ+Nec-1-treated HT-29 cells after 4 h (green: p-MLKL; blue: DAPI). **E** HT-29 (shMLKL) cells stably expressing tamoxifen-inducible MLKL T357E/S358D were pretreated with tamoxifen for 12 h. Phen or Nec-1 or necrosulfonamide (NSA, 1 μM) was added 3 h after the tamoxifen treatment. **F** HT-29 cells were pretreated with Phen or Nec-1 for 1 h and then treated with TRAIL (50 ng/mL) + Smac + z-VAD. The cell lysates were analyzed by western blotting (upper panel), and cell viability was determined by the LDH leakage (middle panel) or the MTT assay (bottom panel). **G** 293 T cells were transfected with Flag-tagged RIPK1 (left panel) or Flag-tagged RIPK3 plasmid (right panel) for 18 h. Phen, Nec-1 or GSK’872 (10 μM) was added 4 h after transfection. The cell lysates were analyzed by immunoblotting. **H** Purified baculovirus-expressed recombinant human RIPK1 kinase domain (amino acids [aa] 1-324) proteins were analyzed for kinase activity by in vitro kinase assays.

In accordance with its protective effect on TNF-mediated necroptosis, 100 μM Phen suppressed the phosphorylation of RIPK1 (Serine 166, autophosphorylation site) in response to TSZ treatment (Fig. 4A, B and Fig. S4A). Inhibition of RIPK1 phosphorylation was also evident in another human cell line, ectopic RIPK3-expressing MDA-MB231 cells, as well as in murine cells, specifically MC-38 cells and MEFs (Fig. 4C and Fig. S4B, C). In addition to their inability to inhibit cell death (see Fig. 3C, D and Fig. S3A, B), ethosuximide and methsuximide did not suppress the phosphorylation of RIPK1 in response to TSZ (Fig. S4D). The phosphorylation of MLKL by RIPK3 ultimately results in the phosphorylation of downstream proteins in the necroptosis signaling pathway, and Phen treatment substantially reduced the TNF-induced phosphorylation of MLKL, which abolished the TNF-induced phosphorylation of MLKL similar to Nec-1 (Fig. 4D, Fig. S4E), whereas GSK’872 (a RIPK3 kinase inhibitor) inhibited only downstream RIPK3 phosphorylation (Fig. S4E). We further used tamoxifen-inducible active MLKL-expressing cells (HT-29 (shMLKL/T357E/S358D)) to induce necroptosis in the absence of upstream signals such as TSZ. As shown in Fig. 4E, Nec-1 and Phen did not inhibit active MLKL-mediated necroptosis, although the MLKL inhibitor necrosulfonamide (NSA) was able to prevent cell death under these conditions (Fig. 4E and Fig. S4F). Since Phen blocks TNF-induced necroptosis, we next tested whether it inhibits TRAIL-induced necroptosis, which is known to occur via a RIPK1-mediated process [34–36]. Notably, Phen not only prevented TNF-α-induced necroptosis but also blocked TRAIL-induced RIPK1 phosphorylation and necroptosis (Fig. 4F and Fig. S4G). The overexpression of RIPK1 or RIPK3 leads to the autophosphorylation of the corresponding protein; therefore, we overexpressed RIPK1 or RIPK3 in 293 cells [37]. Like Nec-1, Phen completely blocked RIPK1 autophosphorylation but did not prevent RIPK3 autophosphorylation, which was abolished by GSK’872, suggesting that Phen is a specific inhibitor of RIPK1 (Fig. 4G). This result was consistently replicated across other cell types, including RIPK1^-/-^/RIPK3-silenced MEFs, RIPK1^-/-^ MEFs and HeLa cells (Fig. S4H–J). To explore whether Phen inhibits RIPK1 kinase activity directly, we purified recombinant human kinase domain of RIPK1 (aa. 1-324) from baculovirus and examined kinase activity with cold ATP. We found that Phen effectively blocked the activation of RIPK1 kinase with similar potency compared to that of Nec-1 (Fig. 4H).

### Phensuximide does not prevent TNF receptor-mediated complex I signaling

**Fig. 5:**
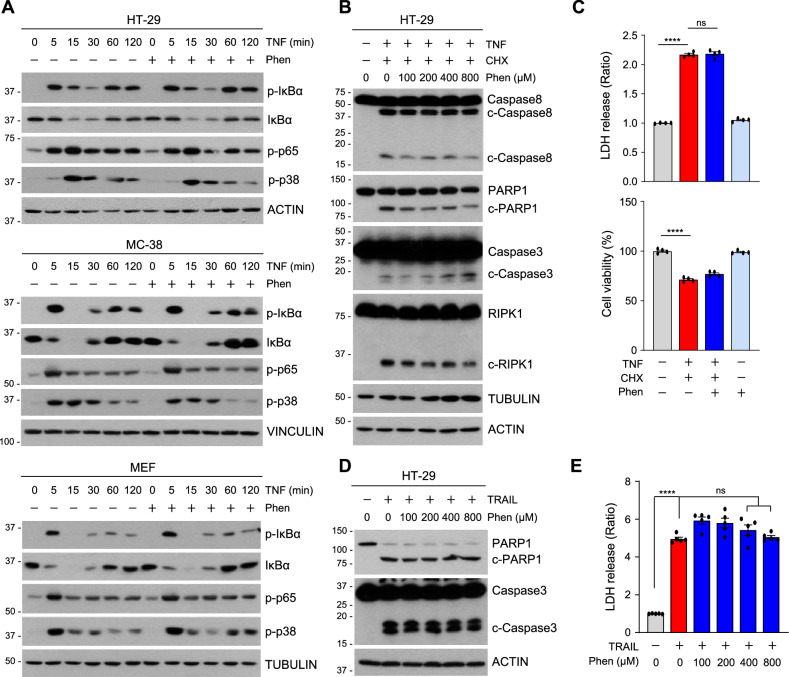
Phensuximide has no effect on TNF-induced NF-κB or MAPK activation. **A** HT-29 cells (upper panel), MC-38 cells (middle panel), or MEFs (bottom panel) were treated with TNF-α for the indicated times in the presence or absence of Phen. The cell lysates were analyzed by immunoblotting. **B**, **C** HT-29 cells were pretreated with the indicated concentrations of Phen or cycloheximide (CHX, 5 μg/mL) for 1 h and then treated with TNF-α. The cell lysates were analyzed by western blotting (B). Cytotoxicity was analyzed by the LDH leakage (upper panel) or the MTT assay (bottom panel) (C). **D**, **E** HT-29 cells were pretreated with the indicated concentration of Phen for 1 h and then treated with TRAIL. The cell lysates were analyzed 6 h after TRAIL treatment by western blotting (D). Cytotoxicity was analyzed by the LDH leakage 12 h after TRAIL treatment (E).

Given the inhibitory effects of Phen on TNFα-induced necroptosis across different cell types and species, characterized by reduced autophosphorylation of RIPK1, we sought to determine whether Phen also hinders other RIPK1-dependent signals from TNF receptor 1 (TNFR1). Upon TNFα binding to TNFR1, the receptor rapidly initiates the formation of what is termed complex I, which facilitates cell survival by activating the NF-κB pathway and the MAP kinase cascade [38]. Therefore, we examined whether Phen affects these pathways. Treatment with TNF-α in the presence or absence of Phen activated both NF-κB and MAPK signaling in a species-independent manner (Fig. 5A). No changes were observed in the ubiquitylation of RIPK1 or its recruitment to TNFR1 following TNF-α treatment, suggesting that the formation of complex I remains unaffected by Phen (Fig. S5A). Activation of downstream TNFR1 signals also induces RIPK1-independent apoptosis in the presence of TNF/cycloheximide (CHX). In this context, Phen neither blocked caspase activation and PARP1 cleavage nor blocked apoptotic cell death, as measured by the MTT and LDH assays, indicating that Phen does not affect the induction of apoptosis (Fig. 5B, C). TRAIL-induced apoptosis was also not affected by the presence of Phen, suggesting that Phen has no influence on receptor-mediated complex formation when it does not rely on RIPK1 kinase activity (Fig. 5D, E and Fig. S5B).

### Inhibition of RIPK1 kinase activity-dependent cell death and the inflammatory response in macrophages by phensuximide

**Fig. 6:**
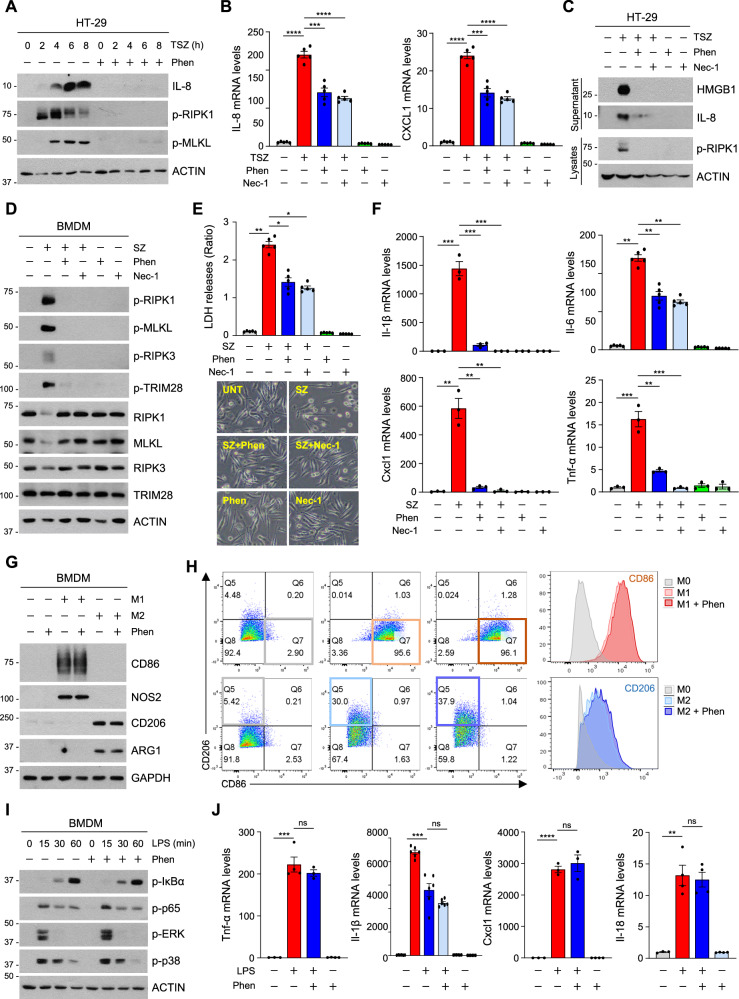
Phensuximide regulates necroptosis-induced immunostimulatory cytokine production. **A** HT-29 cells were pretreated with Phen for 1 h and then treated with TSZ for the indicated times. The cell lysates were analyzed by immunoblotting. **B**, **C** HT-29 cells were treated with TSZ in the presence or absence of Phen or Nec-1. Relative mRNA levels of *IL-8* and *CXCL1* were measured by qPCR (B), and the release of HMGB1 was analyzed by immunoblotting (C). **D–F** BMDMs were pretreated with Phen or Nec-1 for 1 h and then treated with Smac + z-VAD for 6 h. The cell lysates were analyzed by western blotting (D), and cytotoxicity was analyzed by the LDH leakage (E). The mRNA levels of *Il-1β*, *Il-6*, *Cxcl1* and *Tnf-α* were analyzed by qPCR (F). **G**, **H** BMDMs were treated with LPS/IFN-γ (M1) or interleukin-4 (M2) for 24 h in the presence or absence of Phen. The cell lysates were analyzed by western blotting (G), and the expression of CD86 and CD206 in the cells was also evaluated using flow cytometry (H). **I** BMDMs were treated with Phen for 1 h and then treated with LPS for the indicated times. The cell lysates were analyzed by western blotting. **J** BMDMs were treated with Phen for 1 h and then treated with LPS for 4 h. The mRNA levels of *Tnf-α*, *Il-1β*, *Cxcl1* and *Il-18* were analyzed by qPCR.

Previously, we reported that necroptosis activates transcriptional programs to induce the expression of various inflammatory cytokines during the cell death process [39]. Given that RIPK1 has become a promising target in various human inflammatory diseases, we examined whether Phen could inhibit necroptosis-mediated inflammatory cytokine expression. TNF-α-induced necroptosis increased the level of the IL-8 protein in a RIPK1 kinase activity-dependent manner; however, the inhibition of RIPK1 by Phen suppressed these increases (Fig. 6A). Similar to the inhibitory effect of Nec-1, the inhibitory effect of Phen on inflammatory cytokine production occurred at the transcriptional level (Fig. 6B). Necroptosis-mediated plasma membrane rupture is the final step in which cellular components are released as damage-associated molecular patterns (DAMPs), which initiate inflammatory responses, including increases in the levels of inflammatory cytokines. One DAMP, HMGB1, was detected in the supernatant of necroptotic cells [40–42]. However, when Phen was administered, HMGB1 was no longer detected, suggesting the importance of suppressing RIPK1 kinase activity to modulate the inflammatory response (Fig. 6C). Macrophages play pivotal roles in both innate and adaptive immunity and are capable of adopting a diverse spectrum of phenotypes in response to variations in inflammatory microenvironments, each with distinct functions pertaining to immune regulation. We isolated bone marrow-derived macrophages (BMDMs) from the bone marrow and induced necroptosis to investigate the role of Phen. Similar to Nec-1, Phen inhibited RIPK1 kinase activity and suppressed necroptosis in these cells (Fig. 6D, E). As expected, the transcription of inflammatory cytokines under these conditions was abolished by Phen and Nec-1 (Fig. 6F and Fig. S6A). Next, we investigated whether Phen influences macrophage polarization in the context of the inflammatory response, as the polarization states of macrophages play a crucial role in regulating immune homeostasis [43]. Macrophages are mainly classified as pro-inflammatory macrophages (M1-like macrophages) or anti-inflammatory macrophages (M2-like macrophages) when activated. BMDMs differentiate into M1 macrophages when exposed to lipopolysaccharide (LPS)/interferon (IFN)-γ or into M2 macrophages upon stimulation with interleukin (IL)-4 [44, 45]. Phen did not influence macrophage polarization (Fig. 6G, H). An inflammatory mode of cell death termed pyroptosis is another type of cell death that promotes proinflammatory cytokine release from macrophages and has been described as a major driver of septic shock [46]. Canonical pyroptotic death is orchestrated through the assembly of inflammasomes, which are intricate multimolecular complexes activated during host defense against microbial infections, thereby fostering the development of adaptive immune responses [47, 48]. We therefore examined the effect of Phen on pyroptosis. As the activation of Toll-like receptor 4 (TLR4) indirectly promotes the assembly of the inflammasome, thereby amplifying the inflammatory process [49], we first examined whether Phen influences TLR4 signaling. Phensuximide did not alter the activation of MAP kinases or NF-κB, which increases the levels of proinflammatory cytokines under TLR4 stimulation (Fig. 6I, J and Fig. S6B). While fundamental distinctions exist among inflammasomes triggered by various stimuli, canonical inflammasomes typically facilitate caspase-1 activation, leading to pyroptosis, and Phen had no influence on TLR4-mediated inflammasome activation (Fig. S6C). Consistent with this result, pyroptosis has been shown to be independent of the kinase activity of RIPK1, suggesting that Phen only exerts a specific effect on RIPK1 kinase activity-dependent inflammatory diseases.

### Phensuximide is a potential therapeutic candidate for necroptosis-related diseases

**Fig. 7:**
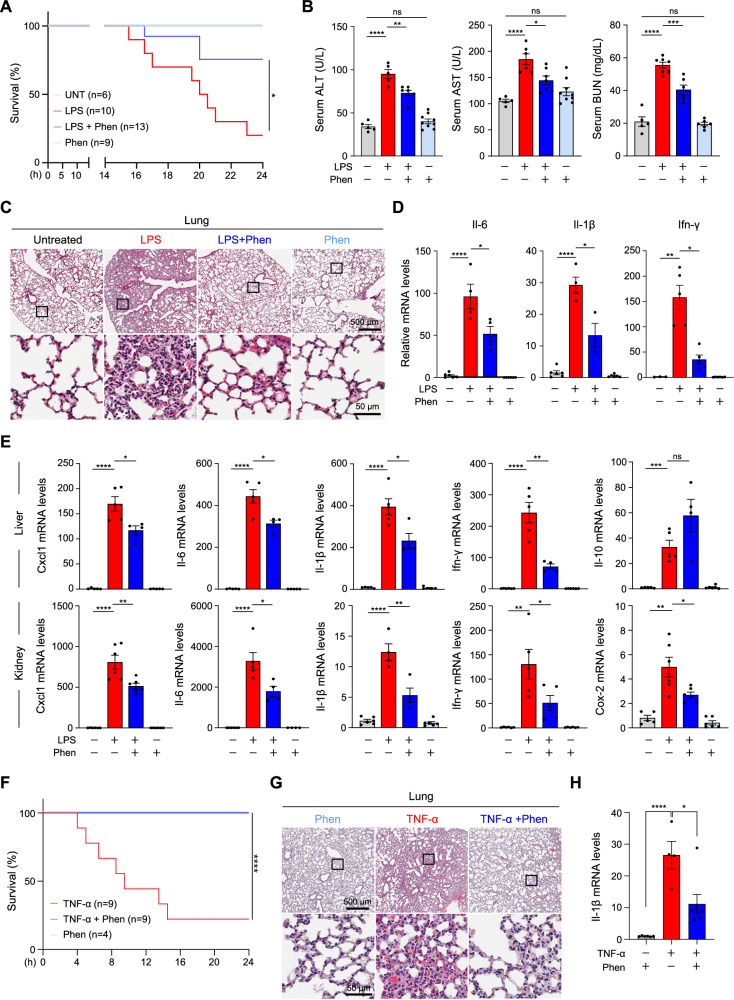
Phensuximide exerts a protective effect on LPS-induced systematic inflammatory response syndrome. **A–E** Eight- to nine-week-old C57BL/6 J mice were pretreated with Phen (50 mg/kg) or vehicle by intraperitoneal injection for 1 h and then intraperitoneally injected with LPS (40 mg/kg). Survival was monitored over a 24-hour period (A). Measurement of ALT, AST and BUN levels in the serum of the mice after 6 h of treatment (B). Representative images of H&E-stained lung tissues after 6 h of treatment are shown. Scale bars = 50 and 500 μm (C). Relative mRNA levels of cytokines in the lung (D), liver (E, upper panel), and kidney (E, bottom panel) after 6 h of treatment. **F–H** Eight- to nine-week-old C57BL/6 J mice were pretreated with Phen (50 mg/kg) or vehicle by intraperitoneal injection for 1 h and then intravenously injected with mTNF-α (750 μg/kg). Survival was monitored over a 24-hour period (F). Representative images of H&E-stained lung tissues are shown. Scale bars = 50 and 500 μm (G). Relative mRNA levels of *Il-1β* in the lung tissues (H). The results are presented as the means $\pm \,$SEMs. Statistical analysis was performed using the unpaired two-tailed Student’s t test.

Our data thus far suggest that Phen is an effective inhibitor of RIPK1 kinase activity; therefore, this inhibitor may be a potential therapeutic drug candidate for necroptosis-related diseases. We next applied Phen to a mouse model of LPS-induced systemic inflammatory response syndrome (SIRS), which is a clinically significant entity resembling septic shock [1, 50–52], to determine the potential of Phen as a novel inhibitor of necroptosis-related diseases. We first confirmed that Phen itself did not induce significant toxicity. The extent of damage to organs was assessed, and the serum alanine aminotransferase (ALT) and aspartate transaminase (AST) levels were measured (Fig. S7A). We intraperitoneally injected Phen or vehicle into the mice starting 1 h before the LPS injection. Pharmacological inhibition of RIPK1 by Phen significantly protected against LPS-induced mortality (Fig. 7A). Serum ALT and AST levels, as well as blood urea nitrogen (BUN) levels, which are markers of cellular damage, were significantly reduced in Phen-treated animals. However, no difference in creatine levels was observed (Fig. 7B and Fig. S7B). Since mortality induced by SIRS is often accompanied by severe organ dysfunction, we proceeded to assess pathological changes occurring in the lungs of this mouse model. After LPS stimulation for 6 hours, the histological examination revealed severe edema and alveolar septal thickening in septic animals, but Phen-treated animals presented substantially reduced lung injury (Fig. 7C). Consistent with the findings of this histological examination, the lung tissues of septic mice presented excessive inflammatory cytokine expression, but decreased inflammatory cytokine expression was observed in Phen-treated animals (Fig. 7D). Kidneys and livers also showed excessive inflammatory cytokine expression (Fig. 7E) that was reduced in the presence of Phen, although hematoxylin- and eosin-stained histological sections of livers and kidneys did not yet reveal remarkable injury at these time points (Fig. S7C). In our LPS-induced septic shock model, Phen provided significant protection, improving both survival and reducing tissue damage and inflammatory markers. To further evaluate its efficacy, we examined another commonly used in vivo septic shock model induced by TNF injection. TNF administration triggers a rapid acute inflammatory response within hours, often leading to high mortality in severe cases [53]. Due to these characteristics, this model is well-suited for rapid drug evaluation and efficient screening of RIPK1 inhibitors. To assess the effect of Phen in TNF-induced septic shock, we administered Phen or vehicle one hour before TNF injection. Within eight hours, over 50% of vehicle-treated mice succumbed to TNF-induced mortality, whereas Phen treatment completely protected against lethality (Fig. 7F). Histological analysis revealed severe edema and alveolar septal thickening in TNF-injected mice, while Phen-treated animals exhibited substantially reduced lung injury (Fig. 7G). Consistent with findings from the LPS-induced model, lung tissues from TNF-injected septic mice showed excessive inflammatory cytokine expression, whereas Phen-treated animals displayed significantly decreased cytokine levels (Fig. 7H and Fig. S7D). These findings suggest that Phen holds promise as an effective therapeutic agent for treating diseases driven by RIPK1.

## Discussion

RIPK1 has garnered attention as a potential drug target for treating a broad range of human diseases due to its recognized role as a key regulator of signaling pathways associated with inflammation and necroptosis [2, 54]. RIPK1 kinase inhibitors have advanced to human clinical trials in pursuit of innovative therapies for inflammatory and neurodegenerative disorders that currently lack efficacious treatments. To date, a few designed drugs are in phase I/II clinical trials; however, no pharmacological inhibitor designed to inhibit RIPK1 kinase activity has currently been approved for clinical use [52, 55]. Drug repositioning (also called drug repurposing) entails the discovery of novel therapeutic purposes for drugs already approved or under investigation, expanding beyond their original medical indications [23]. This approach provides several benefits over developing an entirely new drug for a given indication. The underlying rationale is that the approved or investigational drugs have a similar structure to Nec-1 and a potent docking mode to RIPK1. Here, we identified Phen as a novel and specific RIPK1 kinase inhibitor in both human and murine cells; no effects were observed on RIPK3 kinase activity.

Kinase inhibitors are categorized according to their pharmacodynamic type I, II and III binding modes [56]. Type I kinase inhibitors bind to the ATP-binding site of the kinase and selectively target its active DFG-in conformation. Type II kinase inhibitors target the inactive DFG-out conformation of protein kinases by binding to both the hinge region and the allosteric hydrophobic back pocket, whereas allosteric type III kinase inhibitors also bind the inactive DFG-out state of the kinase domain and exclusively target the hydrophobic back pocket [56]. Nec-1 has an ATP-competitive type III binding model in which the hydroxyl oxygen of Serine 161 within the activation loop of RIPK1 forms a hydrogen bond with the nitrogen atom of the indole ring of Nec-1 [55]. Most selective RIPK1 inhibitors bind to the allosteric and hydrophobic pocket close to the ATP-binding pocket of RIPK1. Due to this property, our identified drug Phen may be specific to only RIPK1 rather than RIPK3, although testing for broader kinome selectivity is still needed. Other members of the succinimide class that contain imide groups, namely, ethosuximide and methsuximide, did not show any inhibitory effect on RIPK1 kinase activity. Although the mechanism of action of Phen remains unclear based on our studies, its effects could also potentially stem from its capacity to hinder the elevation of cyclic AMP and cyclic GMP levels in brain tissue induced by depolarization. However, since commercial drug development efforts have focused primarily on inhibiting RIPK1 kinase activity rather than RIPK3 kinase activity and considering the well-documented pharmacological profile of Phen, Phen would likely be conveniently accessible for clinical use to treat human diseases initiated by aberrant RIPK1 activation. While any of the RIPK1 inhibitors currently in clinical trials exert their effects within the low micromolar range, in this study, we used Phen at concentrations greater than 100 μM in our in vitro system. The reported serum concentration from therapeutic doses of Phen (100 mg, taken 2-3 times daily) ranges from 5.7 μg/mL ( ~ 30 μM) at trough to 20 μg/mL ( ~ 106 μM) or 30 μg/mL ( ~ 160 μM), with patient toxicity not seen until 80 μg/mL [57–59]. This is probably within the inhibitory range for RIPK1 that we used in the in vitro studies without having to push the therapeutic window to levels beyond what is currently being used. Nevertheless, since the side effects of Phensuximide are mild (mostly drowsiness, dizziness, and nausea) [60], it could be considered for drug repositioning as a new drug for RIPK1-mediated inflammatory diseases.

In this study, we utilized both TNF-induced and LPS-induced septic shock models to assess the therapeutic potential of Phensuximide. These models represent distinct but interconnected inflammatory pathways in sepsis. The TNF-induced model is characterized by a rapid inflammatory response, leading to mortality within hours due to RIPK1 kinase-dependent signaling. This model is particularly useful for evaluating RIPK1 inhibitors, as it directly engages TNF receptor-mediated cell death and inflammatory pathways. In contrast, the LPS-induced model mimics a broader, systemic inflammatory response through TLR4 activation, leading to cytokine storm, immune cell infiltration, and progressive multi-organ failure over a longer time frame. While this model also involves RIPK1/RIPK3-mediated necroptosis, its pathophysiology is more complex and not exclusively dependent on RIPK1 activity. Phensuximide demonstrated significant protection in both models, improving survival, reducing tissue damage, and lowering inflammatory cytokine levels. These results support the hypothesis that RIPK1 kinase activity contributes to septic shock pathogenesis, although its precise role remains under investigation.

Previous studies have linked RIPK1 and RIPK3 activation to septic shock, as increased expression of RIPK1, RIPK3, and MLKL has been observed in septic patients [61–63]. However, RIPK1 and RIPK3 also have functions beyond necroptosis, including regulation of inflammatory gene expression and immune responses. While some studies indicate that RIPK1 kinase activity is required for sepsis-induced cytokine production and tissue damage, others report conflicting results, suggesting that RIPK1-regulated gene expression and necroptosis-mediated DAMP release may contribute differently to disease progression [64, 65]. These discrepancies may arise from differences in experimental models, dosing strategies, and endpoint evaluations, highlighting the limitations of current preclinical sepsis models [66]. Given these complexities, Phensuximide’s protective effects in both TNF- and LPS-induced septic shock models suggest its potential as a therapeutic agent for RIPK1-mediated inflammatory diseases, including septic shock. Further studies are needed to clarify its mechanism of action and evaluate its long-term efficacy in sepsis and other inflammatory conditions.

## Materials and methods

### Cell lines and culture conditions

HEK293T, L929, MC-38, HT-29, HeLa, MDA-MB231, MEF, MEF (RIPK1 KO) and HT-29 (shMLKL/T357E/S358D) cells were grown in Dulbecco’s modified Eagle’s medium (DMEM) supplemented with 10% fetal bovine serum (FBS). To generate cell lines stably expressing RIPK3 construct, MDA-MB231 cells were infected with hRIPK3-HA lentivirus. All cells were cultured in 37 °C, 5% CO_2_ incubators. All the cell lines were regularly tested for mycoplasma contamination.

### Primary culture and activation of BMDMs

Bone marrow cells were flushed from dissected femurs of 8–10-week-old C57BL/6 J mice and differentiated in RPMI medium containing 10% fetal bovine serum (FBS), 10 mM HEPES, 2 mM L-glutamine, 10 mM MEM non-essential amino acids solution, and penicillin/streptomycin with 20 ng/mL M-CSF (Peprotech) for 5-7 days. They were cultured in 37 °C, 5% CO_2_ incubators.

### Antibodies and chemical reagents

Antibodies used for immunoblot and immunoprecipitation analysis were as follows: anti-RIPK1 (BD Biosciences, 610458, 1:1000), anti-RIPK1 (Cell signaling Technology, 3493, 1:1000), anti-p-RIPK1(Cell signaling Technology, 65746, 1:1000), anti-RIPK3 (Cell signaling Technology, 13526, 1:2000), anti-p-RIPK3 (S227) (Abcam, ab209384, 1:1000), anti-MLKL (ProteinTech, 66675, 1:2000), anti-p-MLKL (Abcam, ab187091, 1:1000), anti-mouse-p-RIPK1 (Cell Signaling Technology, 31122, 1:1000), anti-mouse-p-RIPK3 (Cell Signaling Technology, 91702, 1:1000), anti-mouse-p-MLKL (Abcam, ab196436, 1:5000), anti-mouse-RIPK3 (ProSci, 2283, 1:2000), anti-β-actin (Santa Cruz, 47778, 1:5000), anti-Vinculin (Sigma Aldrich, V9131, 1:5000), anti-PARP1 (Cell Signaling Technology, 9542, 1:1000), anti-Flag (Sigma Aldrich, F3165, 1:1000), anti-GAPDH (Santa Cruz, 25778, 1:2500), anti-p38 (Cell Signaling Technology, 9212, 1:2000), anti-p-p38 (Cell Signaling Technology, 4511S, 1:2000) anti-p65 (Cell Signaling Technology, 6956, 1:1000), anti-p-p65 (Cell Signaling Technology, 3033, 1:1000), anti-p-ERK (Cell Signaling Technology, 9101S, 1:4000), anti-IκBα (Santa Cruz, 371, 1:1000), anti-p-IκBα (Cell Signaling Technology, 2859, 1:1000), anti-TRIM28 (Cell Signaling Technology, 4123, 1:1000), anti-p-TRIM28 (S473) (BioLegend, 654102, 1:2000), anti-p-TRIM28 (S824) (Cell Signaling Technology, 4127, 1:1000), anti-IL-8 (Proteintech, 17038-1-AP, 1:1000), anti-Caspase-1 (Adipogen, AG-20B-0042, 1:1000), anti-Caspase-3 (Cell Signaling Technology, 9662, 1:1000), anti-Caspase-8 (Cell Signaling Technology, 9746, 1:1000), anti-HMGB1 (Abcam, ab18256, 1:1000), anti-mouse-CD86 (Cell Signaling Technology, 19589, 1:1000), anti-NOS2 (Cell Signaling Technology, 13120, 1:1000), anti-CD206 (Cell Signaling Technology, 24595, 1:5000), anti-ARG1 (Bethyl Laboratories, A700-161, 1:1000), anti-mouse-GSDMD (Abcam, ab225867, 1:1000), anti-Tubulin. Phensuximide, Methsuximide, Ethotoin and Ethosuximide were purchased from MedChemExpress. TNF-α was purchased from R&D Systems. z-VAD-FMK was purchased from ENZO. The SMAC mimetic (LCL-161) was obtained from ApexBio. Necrostatin-1 and lipopolysaccharide (LPS) were purchased from Sigma-Aldrich. NSA and GSK’872 were purchased from Merck. Cycloheximide was from Calbiochem. SytoxOrange Dead Cell Stain was purchased from Thermo Fisher. Polyehylenmine was purchased from Polysciences. GST-TRAIL was purified in the laboratory.

### Immunoblot analysis and immunoprecipitation

For immunoprecipitation, cells were lysed in M2 buffer (20 mM Tris at pH 7, 0.5% NP-40, 250 mM NaCl, 3 mM EDTA, 3 mM EGTA, 2 mM DTT, 0.5 mM PMSF, 20 mM β-glycerol phosphate, 1 mM sodium vanadate, and 1 mg/mL leupeptin). Equal amounts of cell lysates were incubated with the appropriate antibody overnight at 4 °C. The immunocomplex was captured by protein A/G agarose beads for additional 3 h at 4 °C. Bound proteins were removed by boiling in SDS and resolved by SDS-PAGE, and immunoblotting was visualized by enhanced chemiluminescence (Pierce™ ECL Western Blotting Substrate, 32106).

### Plasmid construction and transfection

Flag-RIPK1 and Flag-RIPK3 were generated using LR cloning (Invitrogen, LR clonase). For transient expression, constructs were transfected into cells using polyethylenimine (Polysciences) or Lipofectamine Plus (Invitrogen).

### Cytotoxicity assays

Cell viability was determined using the tetrazolium dye colorimetric test (MTT assay) read at 570 nm. Lactate dehydrogenase (LDH) leakage was quantified using CytoTox 96® Non-Radioactive Cytotoxicity Assay kit (Promega, G1780) according to the manufacturer’s instructions. LDH absorbance was measured at 490 nm. Absorbance was measured using a VersaMax Microplate Reader. Dead cells were detected by uptake of the cell impermeable dye SytoxOrange (Thermo Fisher, S34861) using Lionheart FX automated microscopy (Agilent).

### Immunocytochemistry staining

HT-29 cells were washed twice with DPBS and fixed in 4% paraformaldehyde for 10 min [67]. To stain phospho-MLKL, cells were permeabilized with 0.25% Triton X-100 for 10 min. After incubation in a blocking buffer (10% fetal bovine serum in DPBS) for 30 min, the primary antibody to phospho-MLKL (Abcam, ab187091) was incubated overnight at 4 °C, and antibody (goat anti-rabbit IgG, 1:250, dilution, Invitrogen) was incubated for 1 h at room temperature. A mounting medium containing DAPI (VECTASHIELD, Cat No. H-1200, Vector Laboratories) was used for counterstaining. Representative images were taken by a confocal microscope.

### In vitro kinase assays

For purifying recombinant human GST-RIPK1 (amino acids [aa] 1–324) protein derived from insect cells. To prepare for these proteins, DH10Bac *E. coli* was transformed with pDEST20 to generate recombinant bacmid DNA. Sf9 cells were transfected with purified bacmid DNA. For protein expression, Sf9 cells were infected with baculovirus and after 5 days, cells were collected and lysed with NETN buffer (25 mM Tris-HCl (pH 8.0), 150 mM NaCl, 1 mM DTT, 1% NP-40, 0.1% Triton X-100 and protease inhibitor). The soluble fraction was separated by centrifugation and incubated with glutathione Sepharose 4B beads at 4 °C for 3 h. The protein-bound beads were washed with NETN buffer and eluted with elution buffer (50 mM HEPES (pH 7.5), 100 mM NaCl, 10% glycerol, 40 mM L-glutathione reduced). Purified baculovirus-expressed recombinant human RIPK1 (amino acids [aa] 1–324) were incubated with kinase buffer (100 mM NaCl, 2 mM MgCl_2_, 2 mM MnCl_2_, 1 mM DTT, 20 mM HEPES, 1 µg/mL Leupeptin and 1 X phosphostop) and 500 μM ATP for 30 min at 30 °C. After incubation, samples were boiled and separated by SDS-PAGE, and immunoblotting was visualized by enhanced chemiluminescence (Pierce™ ECL Western Blotting Substrate, 32106).

### Histology

For histology, Lung, liver and kidney from mice were excised and fixed 48 h in 10% formalin solution at 4 °C. Tissues were embedded in paraffin, cut into 4 µm sections (LEICA, HistoCore MULTICUT), dehydrated and deparaffinized via sequential addition of xylene, 100% ethanol, 95% ethanol, 85% ethanol, and 70% ethanol. The sections were then washed in distilled water and stained with Harris’ Hematoxylin (Electron Microscopy Sciences, 26041-06) and Eosin (Sigma Aldrich, 318906). Mounting solution (Thermo Fisher Scientific, SP15-500) was dropped on the samples and dried at 25 °C for 12 h.

### Flow cytometry

Cell suspensions were stained on ice for 20 min in the dark with various combinations of the following fluorochrome-conjugated antibodies: CD11b (BioLegend, 101206), F4/80 (BioLegend, 123128), CD86 (BioLegend, 105012), CD206 (BioLegend, 141706), CD45 (BioLegend, 103114), Zombie (BioLegend, 77184) and were used according to the manufacturer’s instructions [68].

### Quantitative RT-PCR

RNA quantification was performed as described previously with slight modifications [69]. RNA was extracted using the TRIzol reagent (Life Technologies, 15596018). Total 1 μg of RNA from each sample was used for cDNA synthesis using MMLV reverse transcriptase (Promega, M1705). Equal amounts of cDNA product were used in real-time PCR with GoTaq® qPCR Master Mix (Promega, A6002). Gene expression was normalized to that of actin. Real-time PCR was performed on CFX Connect™. The oligonucleotides are listed in Table S1 (Supporting information).

### Serum parameter measurements

Mice serum samples were obtained from the peripheral blood by centrifugation for 10 min at 6300 *g*, 4 °C. Serum levels of alanine aminotransferase, aspartate aminotransferase, blood urea nitrogen and Creatinine were measured by FUJI DRI-CHEM 3500 s (FUJI PHOTO FILM CO. LTD.) following the manufacturer’s protocols.

### LPS- and TNF-induced SIRS model

Phensuximide (Sino Biological) was dissolved in a solvent consisting of 6.7% DMSO, 2% Tween-80, and 40% PEG-300 in PBS. 8-9-week-old C57BL/6 J mice were pretreated intraperitoneally with Phen (50 mg/kg) or vehicle for 1 h prior to the administration of either LPS (40 mg/kg, diluted in PBS) intraperitoneally or mTNF-α (750 μg/kg, diluted in PBS) intravenously. Survival was monitored over a 24 h period, and mice were promptly sacrificed when mice died. All animals were maintained in specific pathogen-free animal facilities in the Laboratory Animal Research Center of Ajou University and were maintained according to the guidelines of the Institutional Animal Care and Use Committee, who approved all animal procedures (2023-0003).

### Structural similarity calculation

To calculate structural similarity, the simplified molecular-input line-entry system (SMILES) molecular representation for each compound was derived from DrugBank database and converted into an extended-connectivity fingerprint (ECFP) using RDKit. Jaccard similarity, a measure of similarity between two asymmetric binary vectors, was calculated as a chemical similarity score. The resulting index ranges from 0 to 1, where 0 indicates no shared elements between compounds, and 1 represents identical compounds.

### Molecular docking and dynamics simulation study

To study the interactions between potential RIPK1 inhibitors and RIPK1, protein preparation was undertaken using the ADFR software suite applied to the protein structure derived from PDB ID 4ITH. Molecular docking simulations were executed employing AutoDock Vina version 1.2.3. The center of the docking grid was defined by the 3D coordinates of the ligand present in the 4ITH structure, with grid dimensions set uniformly to 20 units for size_x, size_y, and size_z. The simulations were conducted with an exhaustiveness level of 32, and all other parameters were maintained at default settings. Subsequent molecular dynamics simulations were performed over a 10 ns period utilizing GROMACS version 2023.1. The outcomes of the molecular dynamics’ simulation were analyzed using MDAnalysis to investigate the interactions and dynamics between the protein and the ligand.

### Statistical analysis

Each experiment was repeated three times or more. Statistical analyses were conducted using unpaired Student’s *t*-test or two-way ANOVA. *P*-values below 0.05 were considered significant in the following manner: *p < 0.05, **p < 0.01, ***p  < 0.001, ****p < 0.0001. ns, not significant.

## Supplementary information

Supplementary Figures

Supplementary Figure Legends

Supplementary TableS1

Kim et al_uncropped Image

## Data Availability

The data generated or analyzed during this study are included in this published article and its supplementary information files.
