# Supplementary material for: FDA-approved phensuximide inhibits RIPK1-dependent immunogenic cell death
Source: Cell Death Dis. 2025 Jun 2;16(1):426. doi: 10.1038/s41419-025-07754-2 (PMC12130204; doi:10.1038/s41419-025-07754-2)

Supplementary Figure 1

A

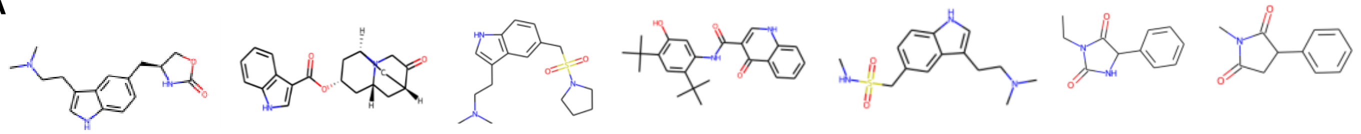

1 Zolmitriptan

2 Dolasetron

3 Almotriptan

4 Ivacaftor

5 Sumatriptan

6 Ethotoin

7 Phensuximide

B

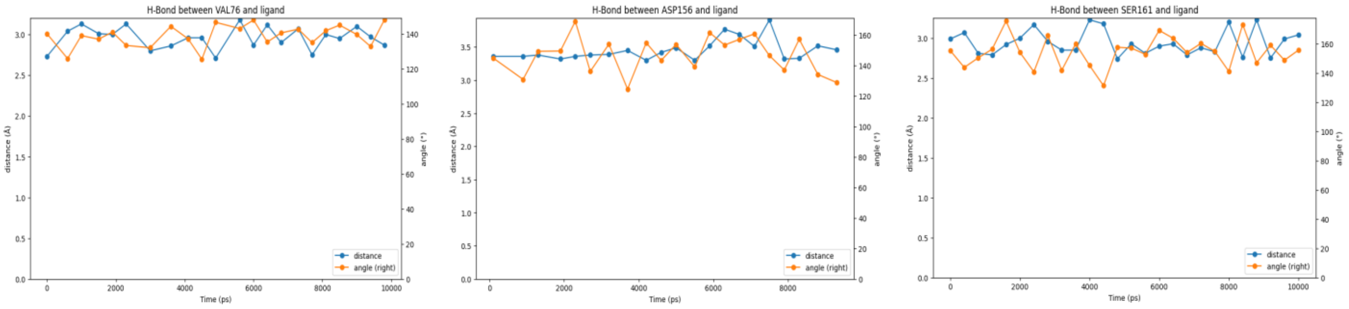

C

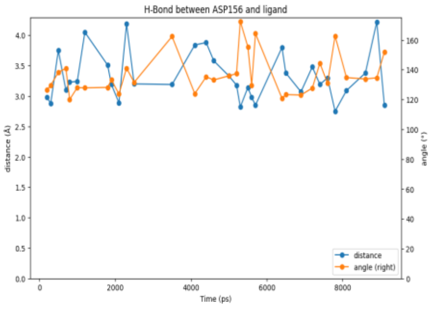

Supplementary Figure 2

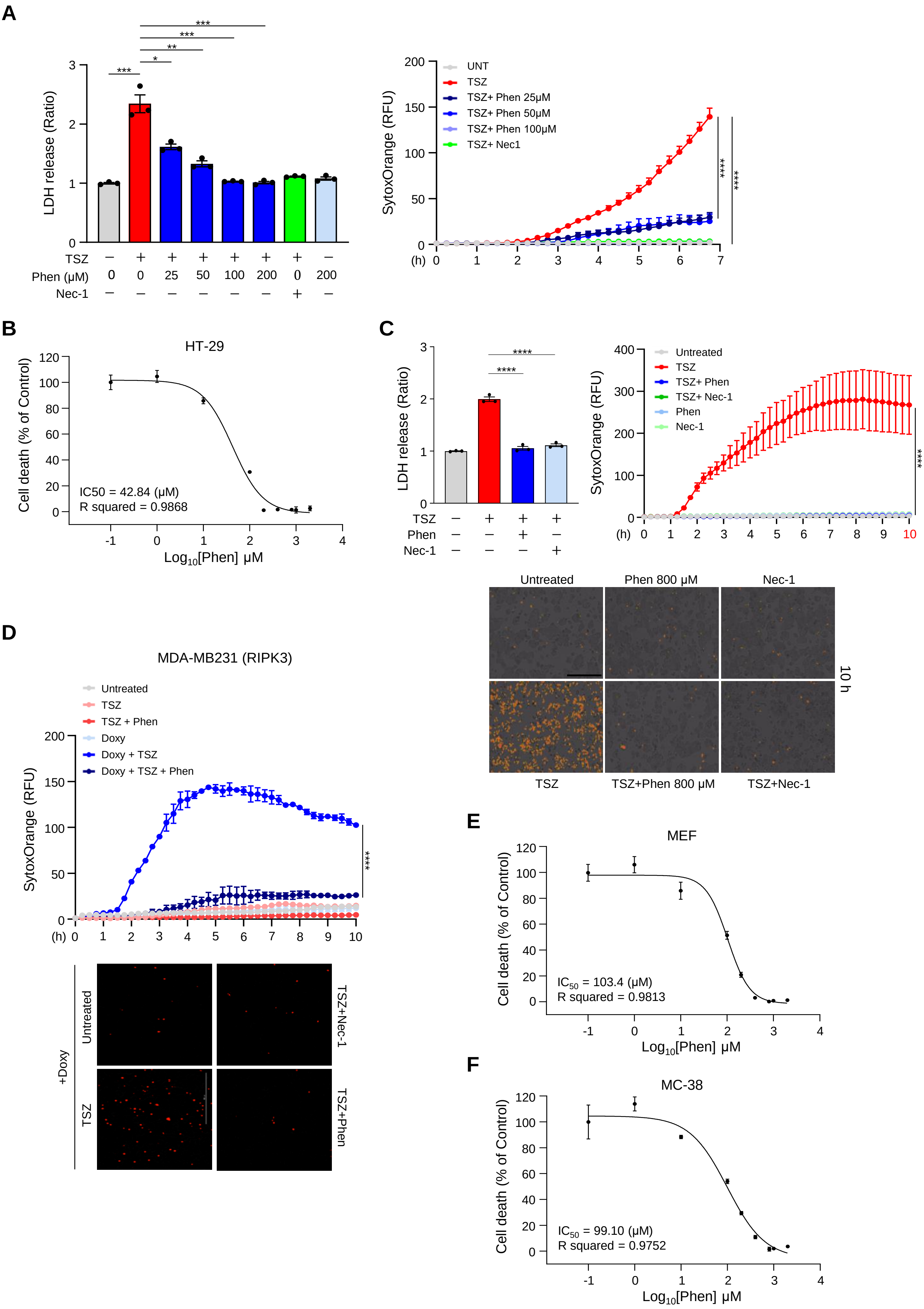

Supplementary Figure 3

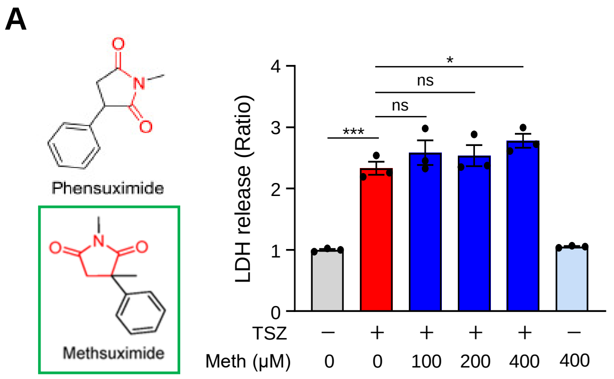

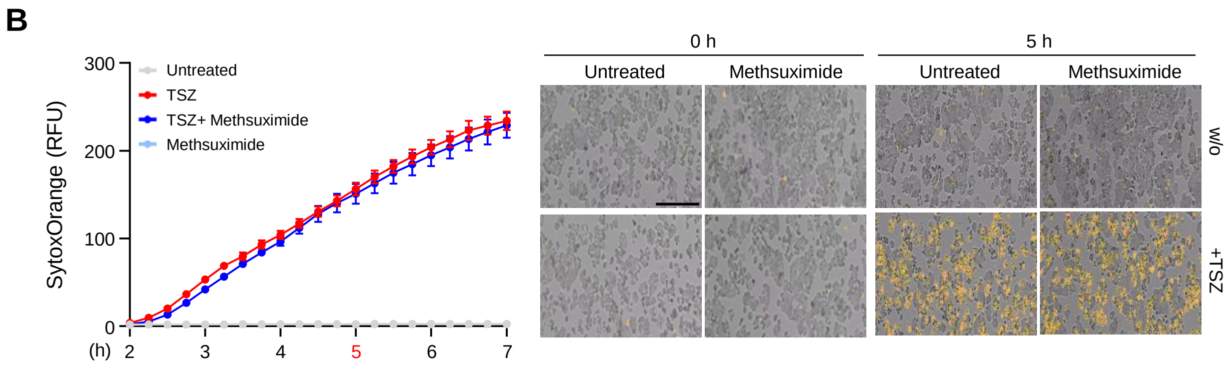

Supplementary Figure 4

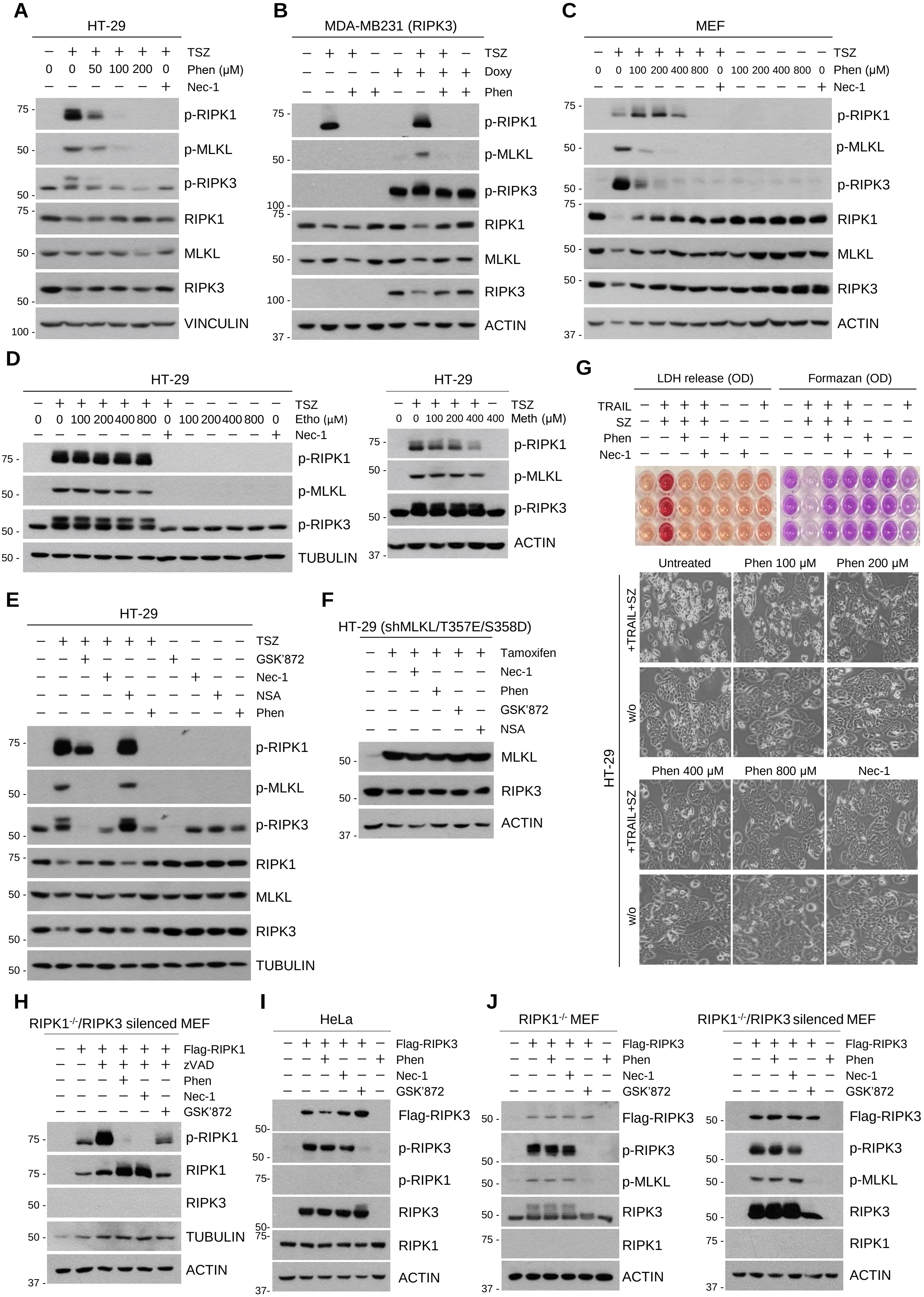

Supplementary Figure 5

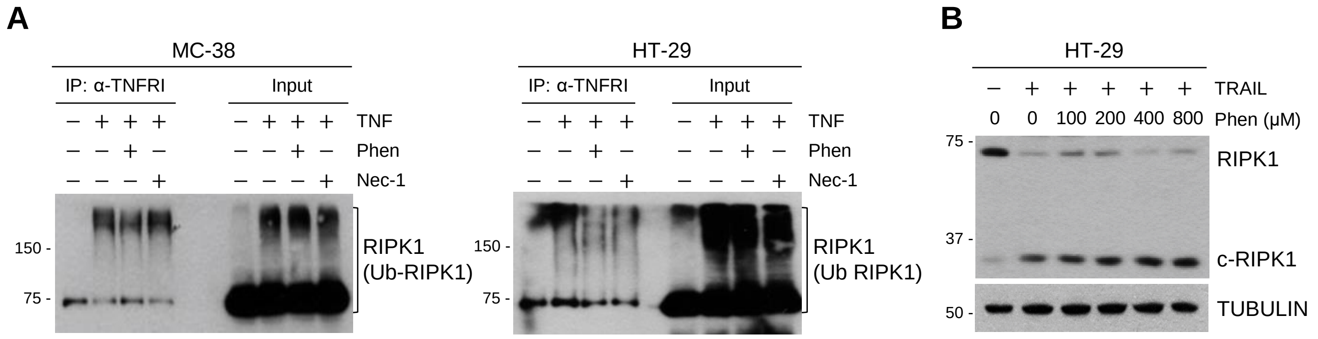

Supplementary Figure 6

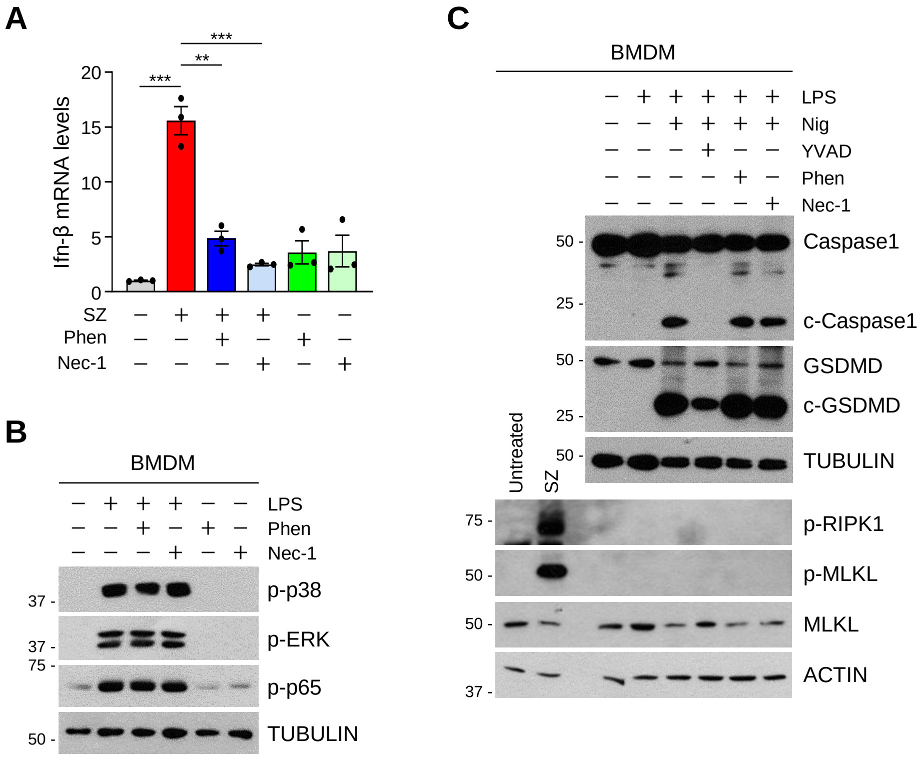

Supplementary Figure 7

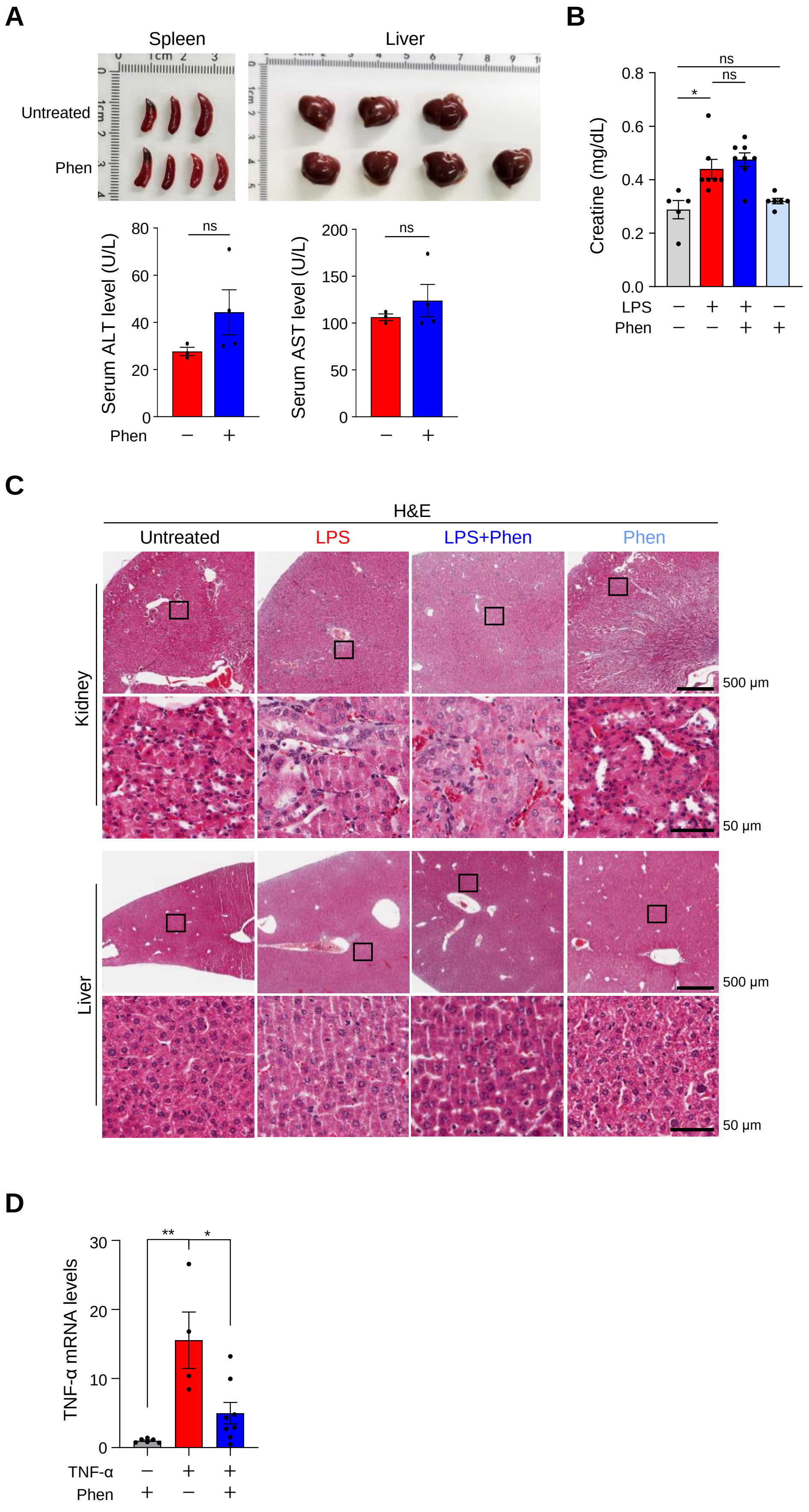

Supplement: Supplementary file 1 — Supplementary Figures [file 41419_2025_7754_MOESM1_ESM.pdf]
