# Supplementary material for: FDA-approved phensuximide inhibits RIPK1-dependent immunogenic cell death
Source: Cell Death Dis. 2025 Jun 2;16(1):426. doi: 10.1038/s41419-025-07754-2 (PMC12130204; doi:10.1038/s41419-025-07754-2)
Supplement: Supplementary file 2 — Supplementary Figure Legends [file 41419_2025_7754_MOESM2_ESM.docx]

**SUPPLEMENTARY FIGURE LEGENDS**

**Figure S1. Identification of highly similar drugs to Necrostatin-1.**

(A) Chemical structures of the top 7 drugs ranked in terms of similarity to Nec-1.

(B and C) Hydrogen bond dynamics simulation of Nec-1 (B) and Phen (C).

**Figure S2. Phensuximide protects cells from TNF-mediated necroptosis.**

(A) HT-29 cells were pretreated with the indicated concentrations of Phen or Nec-1 for 1 h and then treated with TSZ. Cytotoxicity was analyzed by the LDH leakage at 4 h posttreatment (left panel) or measured by the fluorescence intensity of Sytox Orange using a Lionheart FX automated microscope (right panel).

(B) Evaluation of IC50 of Phen in TSZ-induced cell death. HT-29 cells were pretreated with various concentrations of Phen and then treated with TSZ to measure the inhibitory effect. Cell cytotoxicity was measured by LDH releases.

(C) HT-29 cells were pretreated with Phen (800 μM) or Nec-1 for 1 h and then treated with TSZ. Cytotoxicity was analyzed by the LDH leakage at 4 h posttreatment (left panel) or the fluorescence intensity of Sytox Orange (middle panel). Representative images of cell death were taken at 10 h (right panel).

(D) Stable MDA-MB231 cells expressing doxycycline-inducible RIPK3 were treated with TSZ in the presence or absence of Phen. Cytotoxicity was measured by the fluorescence intensity of Sytox Orange.

(E & F) Evaluation of IC50 of Phen in TSZ-induced cell death. MC-38 (E) and MEF (F) cells were pretreated with various concentrations of Phen and then treated with TSZ to measure the inhibitory effect. Cell cytotoxicity was measured by LDH releases.

**Figure S3. Methsuximide has no inhibitory effect on TNF-mediated necroptosis.**

(A) Chemical structures of Phen and methsuximide (left panel). HT-29 cells were pretreated with the indicated concentrations of methsuximide for 1 h and then treated with TSZ for 5 h. Cytotoxicity was determined by the LDH assay (right panel).

(B) HT-29 cells were pretreated with methsuximide (400 μM) for 1 h and then treated with TSZ. Cytotoxicity was measured by analyzing the fluorescence intensity of Sytox Orange, and representative images of cell death were taken at 5 h.

**Figure S4. Phensuximide efficiently blocks RIPK1 S166 autophosphorylation during necroptosis.**

(A) HT-29 cells were pretreated with the indicated concentrations of Phen or Nec-1 for 1 h and then treated with TSZ for 4 h. The cell lysates were analyzed via western blotting.

(B) MDA-MB231 cells stably expressing doxycycline-inducible RIPK3 were treated with TSZ in the presence or absence of Phen for 3 h. The cell lysates were analyzed by western blotting.

(C) MEFs were pretreated with the indicated concentrations of Phen or Nec-1 for 1 h and then treated with TSZ. The cell lysates were analyzed by western blotting.

(D) HT-29 cells were pretreated with the indicated concentrations of ethosuximide (left panel) or methsuximide (right panel) for 1 h and then treated with TSZ for 4 h. The cell lysates were analyzed via western blotting.

(E) HT-29 cells were pretreated with GSK’872, Nec-1, NSA or Phen for 1 h and then treated with TSZ for 4 h. The cell lysates were analyzed by western blotting.

(F) HT-29 (shMLKL) cells stably expressing tamoxifen-inducible MLKL T357E/S358D were pretreated with tamoxifen for 12 h. Phen, Nec-1, NSA or GSK’872 was added 3 h after the tamoxifen treatment. The cell lysates were analyzed by western blotting.

(G) HT-29 cells were pretreated with Phen for 1 h and then treated with TRAIL + SZ for 6 h. Cytotoxicity was analyzed by the LDH leakage or the MTT assay (upper panel). Images are representative of at least three independent experiments (bottom panel).

(H) RIPK1^-/-^/RIPK3-silenced MEFs were transfected with a Flag-tagged RIPK1 plasmid for 18 h. Phen, Nec-1, GSK’872 or zVAD were added 4 h after transfection. The cell lysates were analyzed by immunoblotting.

(I) HeLa cells were transfected with a Flag-tagged RIPK3 plasmid for 18 h. Phen, Nec-1 or GSK’872 was added 4 h after transfection. The cell lysates were analyzed by immunoblotting.

(J) RIPK1^-/-^ MEFs (left panel) or RIPK1^-/-^/RIPK3-silenced MEFs (right panel) were transfected with a Flag-tagged RIPK3 plasmid for 18 h. Phen, Nec-1 or GSK’872 was added 4 h after transfection. The cell lysates were analyzed by western blotting.

**Figure S5. Phensuximide does not affect RIPK1 recruitment to the TNF receptor in response to TNF-α.**

(A) MC-38 (left panel) or HT-29 cells (right panel) were pretreated with Phen or Nec-1 for 1 h and then treated with TNF-α for 10 mins. The cells were harvested and immunoprecipitated with TNFR1 antibodies.

(B) HT-29 cells were pretreated with the indicated concentrations of Phen for 1 h and then treated with TRAIL for 6 h. The cell lysates were analyzed by western blotting.

**Figure S6. Phensuximide reduces cytokine production in BMDMs.**

(A) BMDMs were pretreated with Phen or Nec-1 for 1 h and then treated with SZ for 6 h. Relative mRNA levels of *Ifn-β* were analyzed by qPCR.

(B) BMDMs were treated with LPS in the presence or absence of Phen or Nec-1 for 15 min.

(C) BMDMs were treated with LPS, followed by YVAD, Phen, or Nec-1 three hours later. One hour after that, Nigericin was added and incubated for 15 minutes. The cell lysates were then analyzed by western blotting.

**Figure S7. LPS-induced systemic inflammatory response syndrome is attenuated by phensuximide.**

(A) Mice were injected with PBS or Phen. Liver and spleen tissues were harvested from the animals after 24 h (upper panel). ALT and AST levels in the serum of mice treated with or without Phen for 24 h were measured (bottom panel).

(B & C) Eight- to nine-week-old C57BL/6J mice were pretreated with Phen (50 mg/kg) or vehicle by intraperitoneal injection for 1 h and then intraperitoneally injected with LPS (40 mg/kg). Measurement of creatine levels in the serum of the mice after 6 h of LPS treatment (B). Representative images of H&E-stained kidney (upper panel) or liver (bottom panel) tissues are shown (C). Scale bars = 50 and 500 μm.

(D) Eight- to nine-week-old C57BL/6J mice were pretreated with Phen (50 mg/kg) or vehicle by intraperitoneal injection for 1 h and then intravenously injected with mTNF-α (750 μg/kg). Relative mRNA levels of *Tnf-α* were measured in the lung tissues.
