# Supplementary material for: FDA-approved phensuximide inhibits RIPK1-dependent immunogenic cell death
Source: Cell Death Dis. 2025 Jun 2;16(1):426. doi: 10.1038/s41419-025-07754-2 (PMC12130204; doi:10.1038/s41419-025-07754-2)
Supplement: Supplementary file 3 — Supplementary TableS1 [file 41419_2025_7754_MOESM3_ESM.docx]

**Supplementary Table**

Table S1. Primer sequences used in qPCR

| Species | Gene name |  | Sequence |
| --- | --- | --- | --- |
| Human | IL-8 | F | TCTGCAGCTCTGTGTGAAGG |
|  |  | R | AATTTCTGTGTTGGCGCAGT |
|  | CXCL1 | F | AGGGAATTCACCCCAAGAAC |
|  |  | R | TGGATTTGTCACTGTTCAGCA |
|  | GAPDH | F | GGAGCGAGATCCCTCCAAAAT |
|  |  | R | GGCTGTTGTCATACTTCTCATGG |
| Mouse | Il-1β | F | TGCCACCTTTTGACAGTGATG |
|  |  | R | AAGGTCCACGGGAAAGACAC |
|  | Il-6 | F | TCCAGTTGCCTTCTTGGGAC |
|  |  | R | GTACTCCAGAAGACCAGAGG |
|  | Il-10 | F | TTACTGACTGGCATGAGGATCA |
|  |  | R | AAGGAGTCGGTTAGCAGTATGT |
|  | Il-18 | F | GACAGCCTGTGTTCGAGGAT |
|  |  | R | TGGATCCATTTCCTCAAAGG |
|  | Tnf-α | F | CGAGTGACAAGCCTGTAGCC |
|  |  | R | ACAAGGTACAACCCATCGGC |
|  | Ifn-β | F | AACTATAAGCAGCTCCAGCTC |
|  |  | R | CTTGGATGGCAAAGGCAGTG |
|  | Ifn-γ | F | CGGCACAGTCATTGAAAGCC |
|  |  | R | TGCATCCTTTTTCGCCTTGC |
|  | Cxcl1 | F | TCCAGAGCTTGAAGGTGTTGCC |
|  |  | R | AACCAAGGGAGCTTCAGGGTCA |
|  | Cox-2 | F | TTCAACACACTCTATCACTGGC |
|  |  | R | AGAAGCGTTTGCGGTACTCAT |
|  | Gapdh | F | GGAGCCAAAAGGGTCATCAT |
|  |  | R | GTGATGGCATGGACTGTGGT |
