# Supplementary material for: FDA-approved phensuximide inhibits RIPK1-dependent immunogenic cell death
Source: Cell Death Dis. 2025 Jun 2;16(1):426. doi: 10.1038/s41419-025-07754-2 (PMC12130204; doi:10.1038/s41419-025-07754-2)

Figure 4A

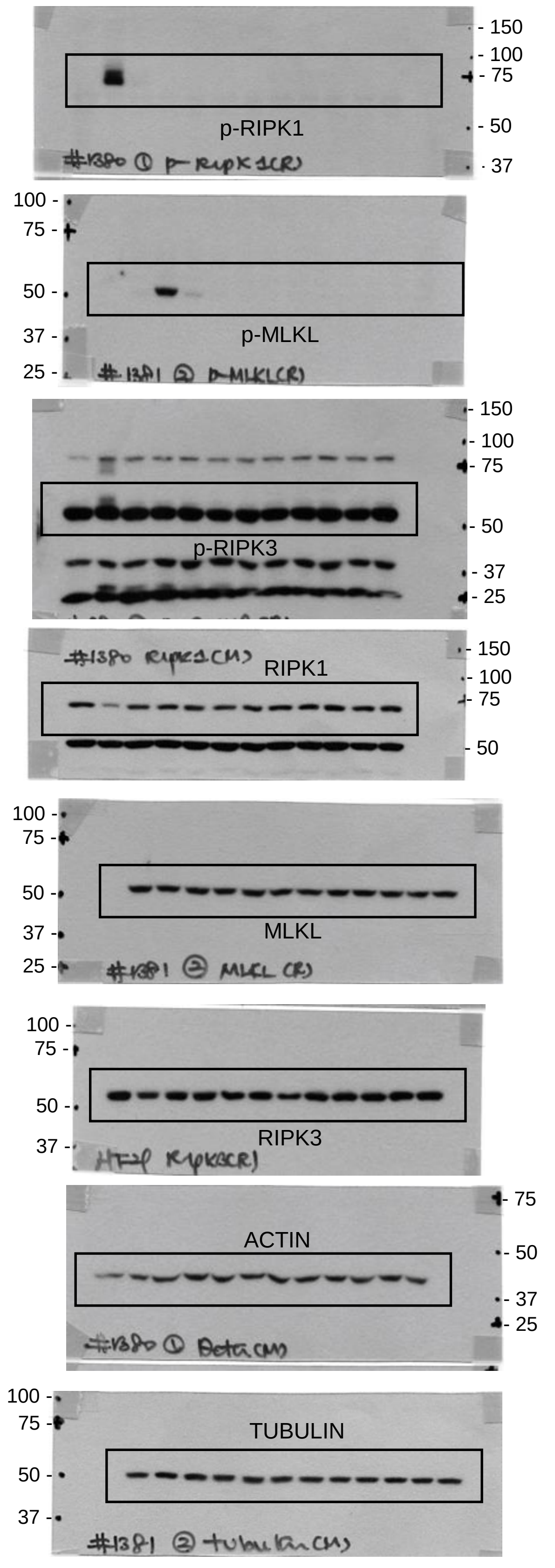

Figure 4B

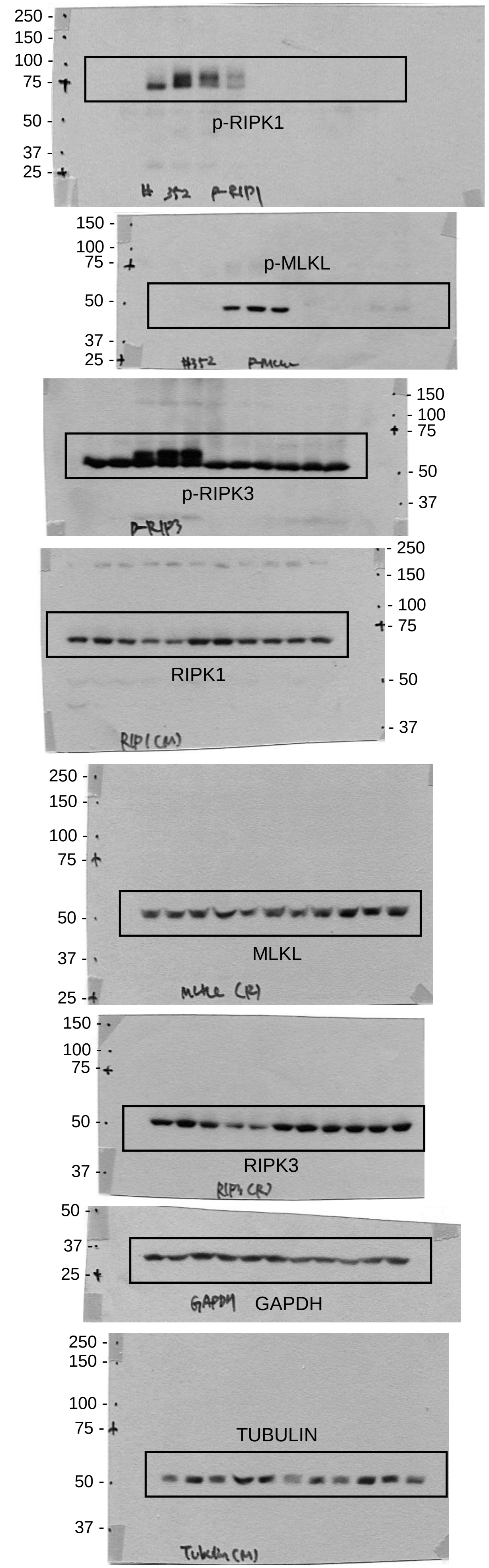

Figure 4C

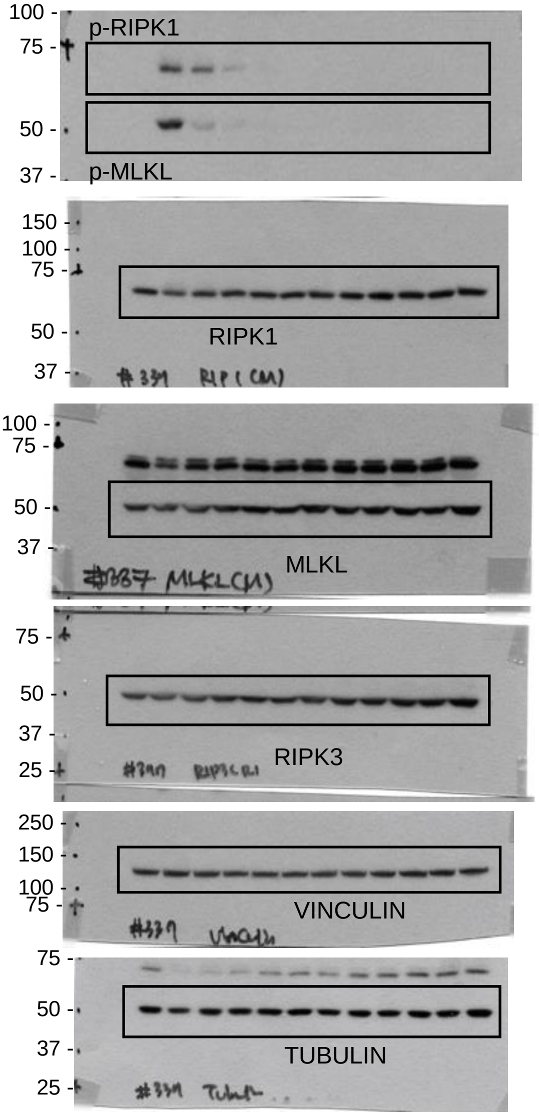

Figure 4F

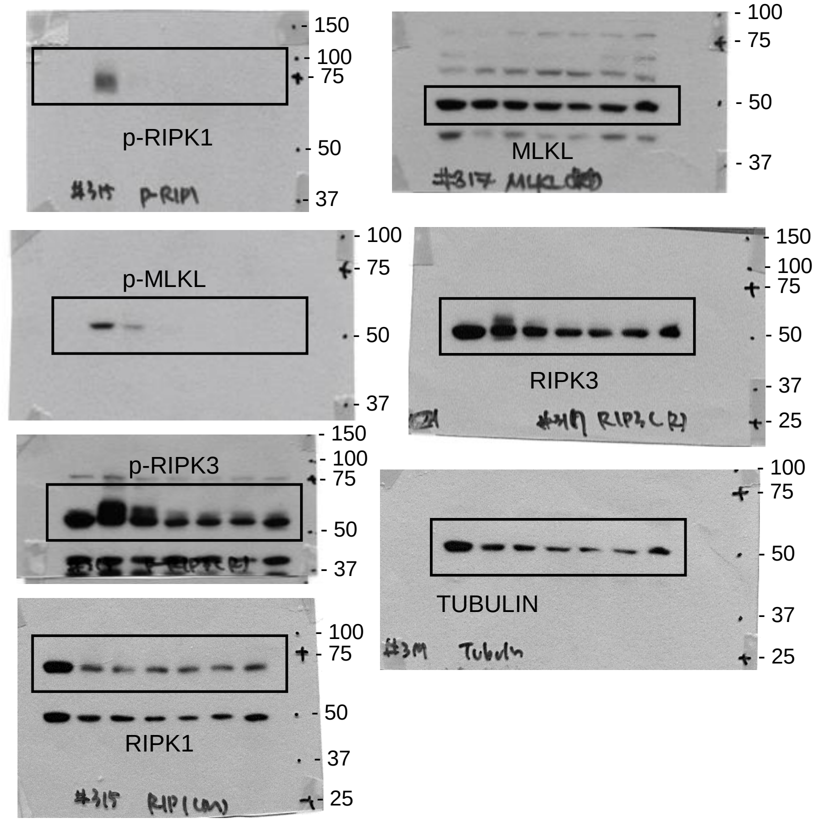

Figure 4G (left)

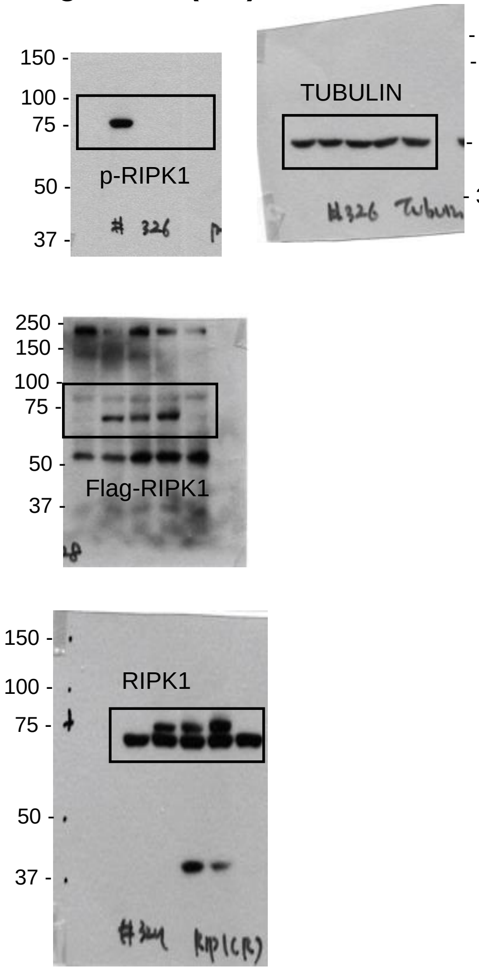

Figure 4G (right)

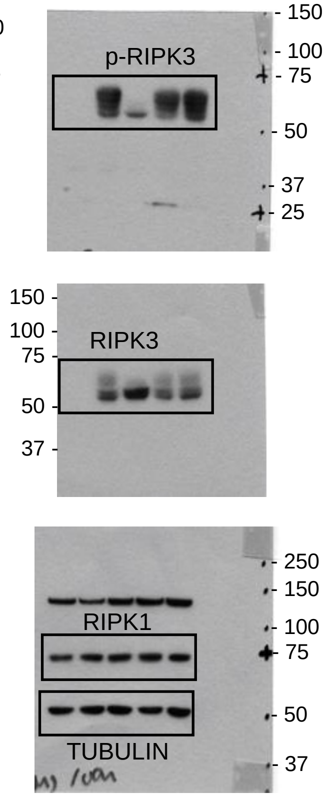

Figure 4H

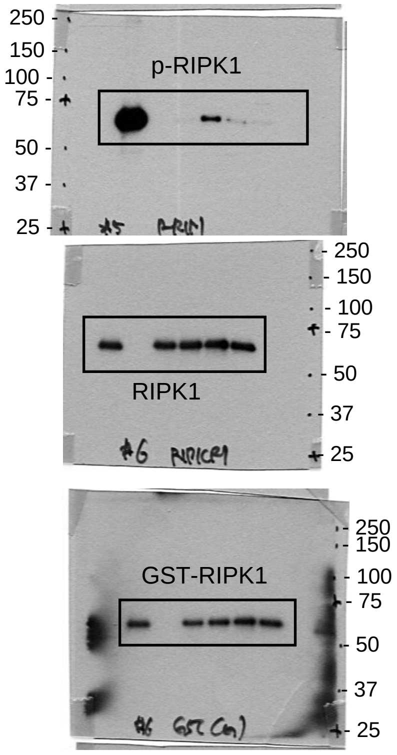

Figure 5A (HT-29)

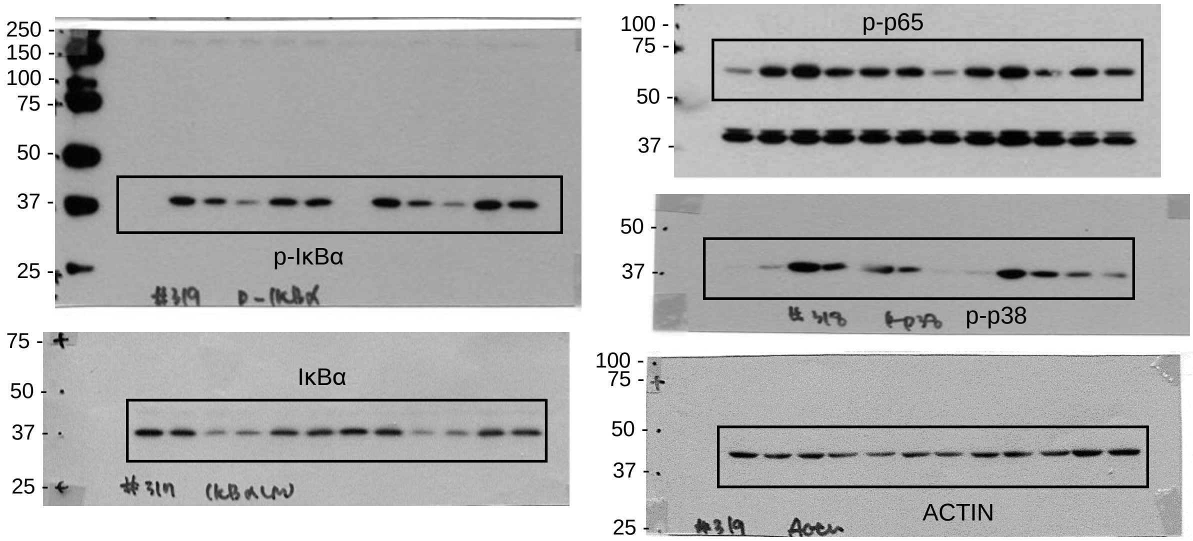

Figure 5A (MC-38)

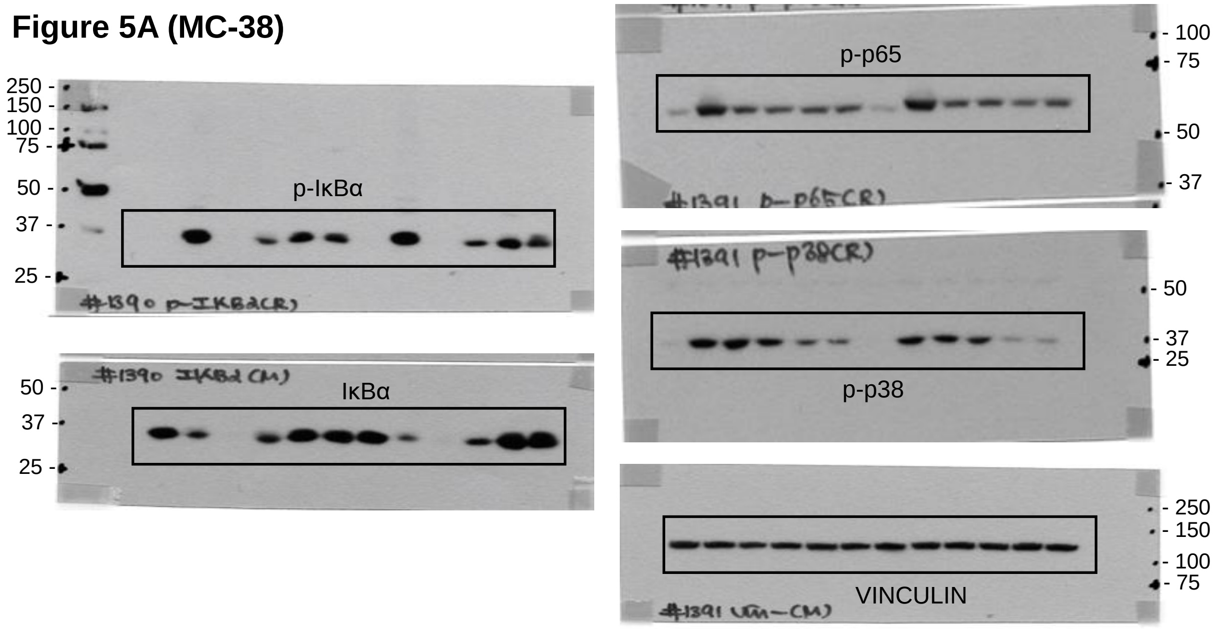

Figure 5A (MEF)

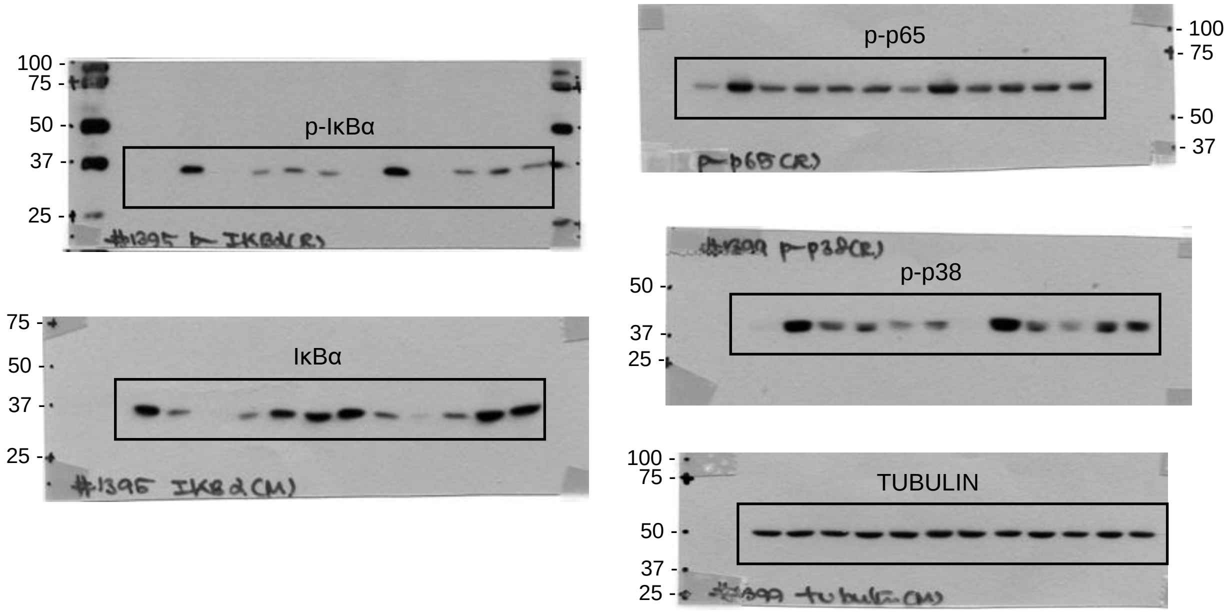

Figure 5B

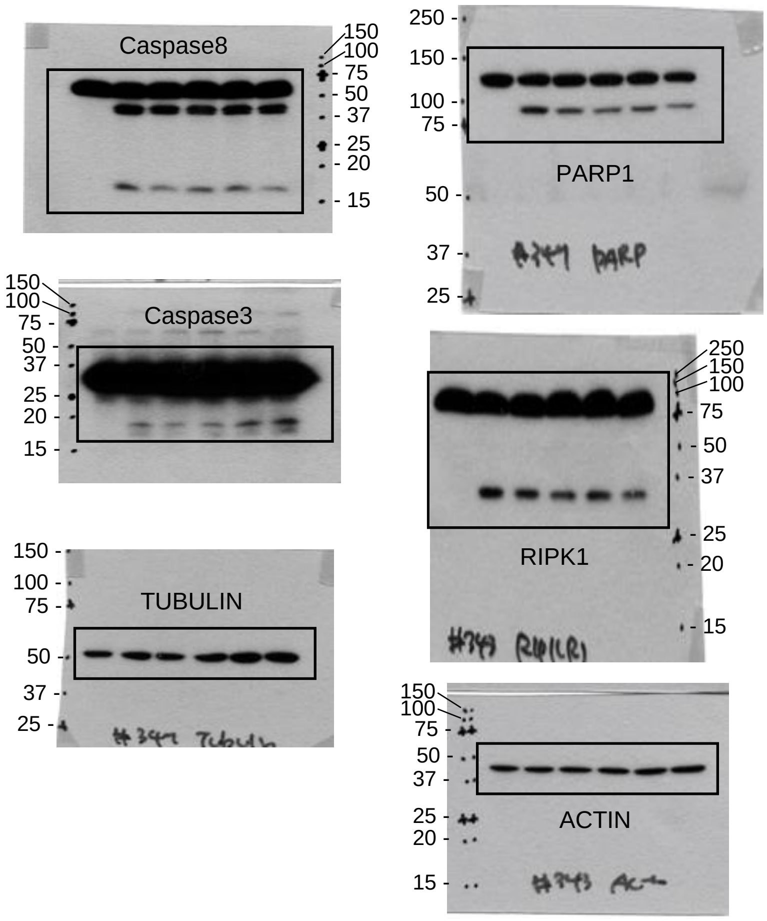

Figure 5D

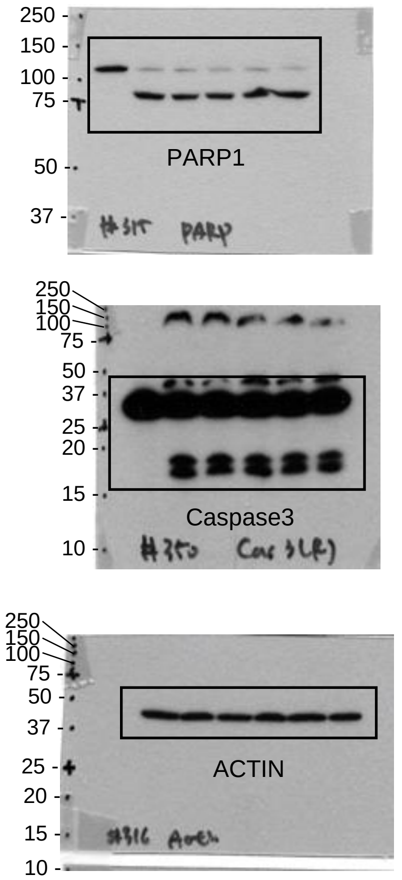

Figure 6A

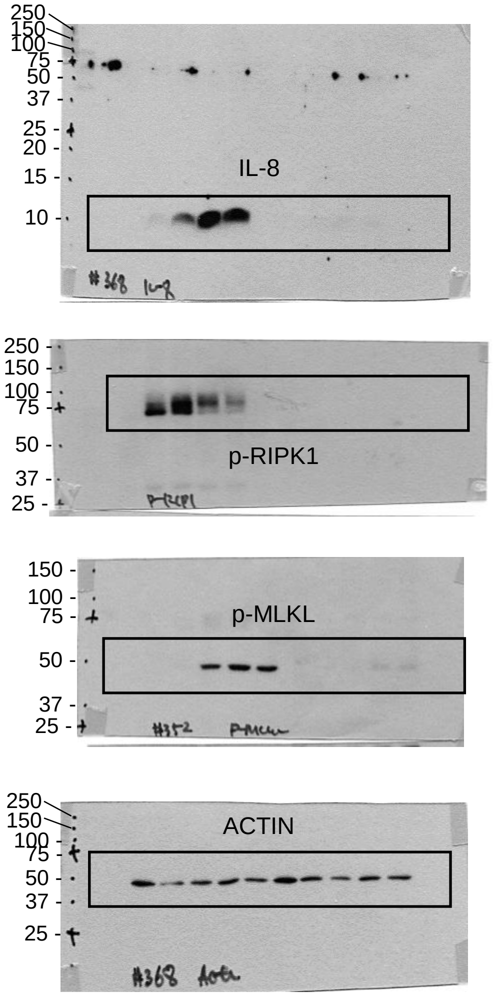

Figure 6C

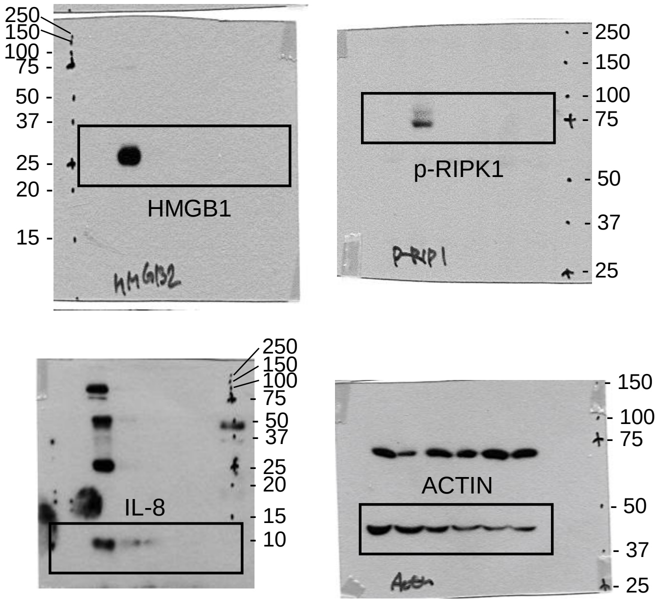

Figure 6D

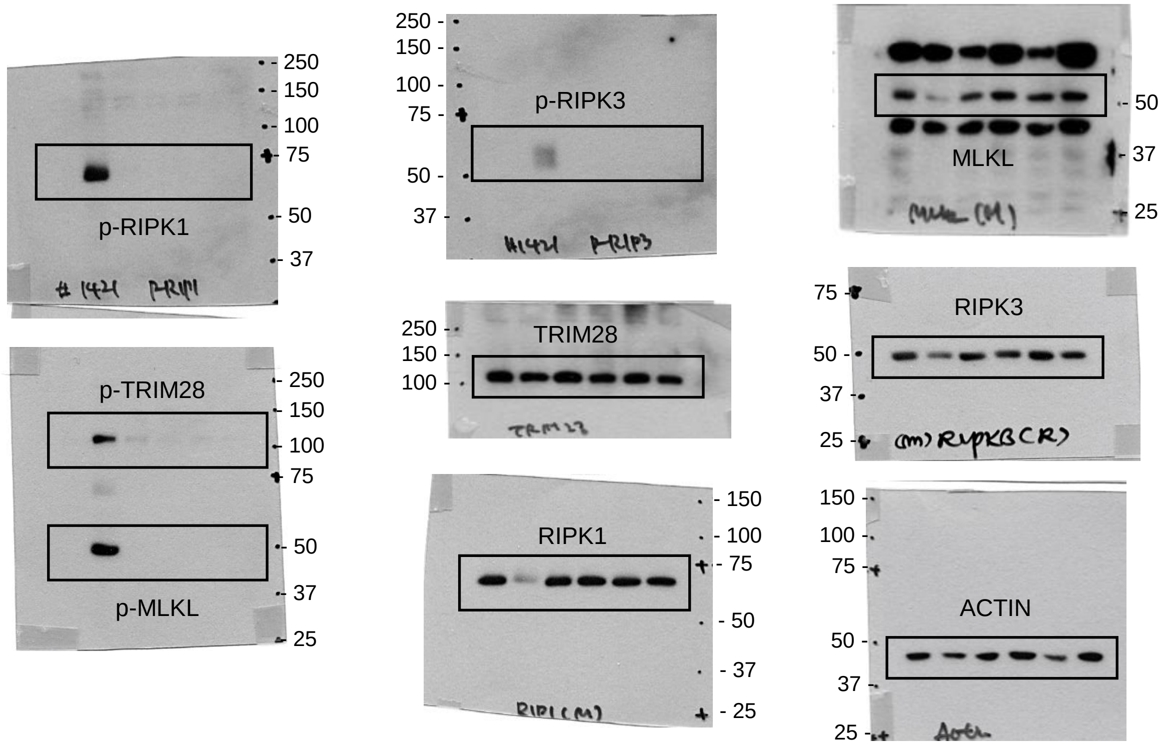

Figure 6G

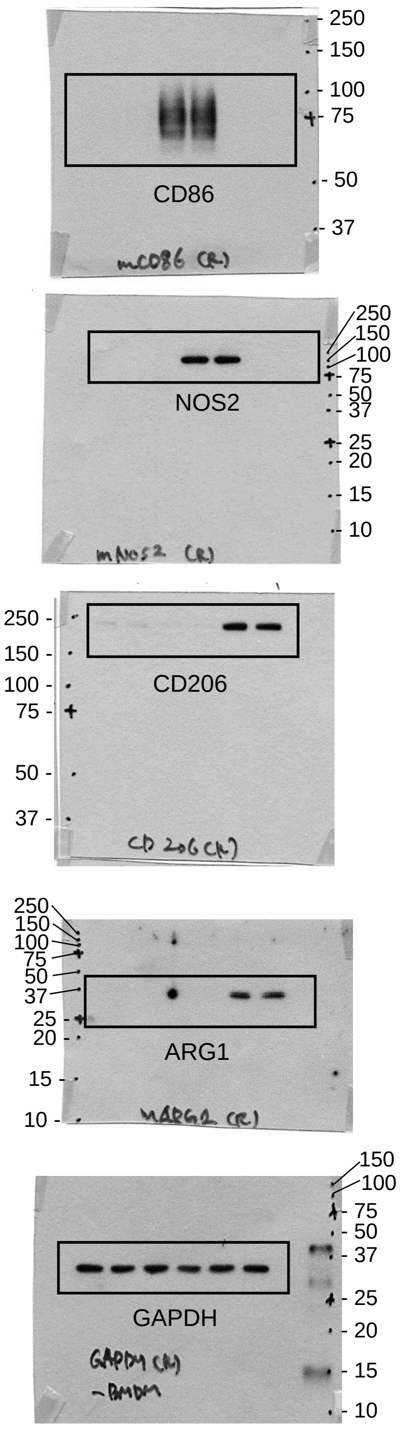

Figure 6I

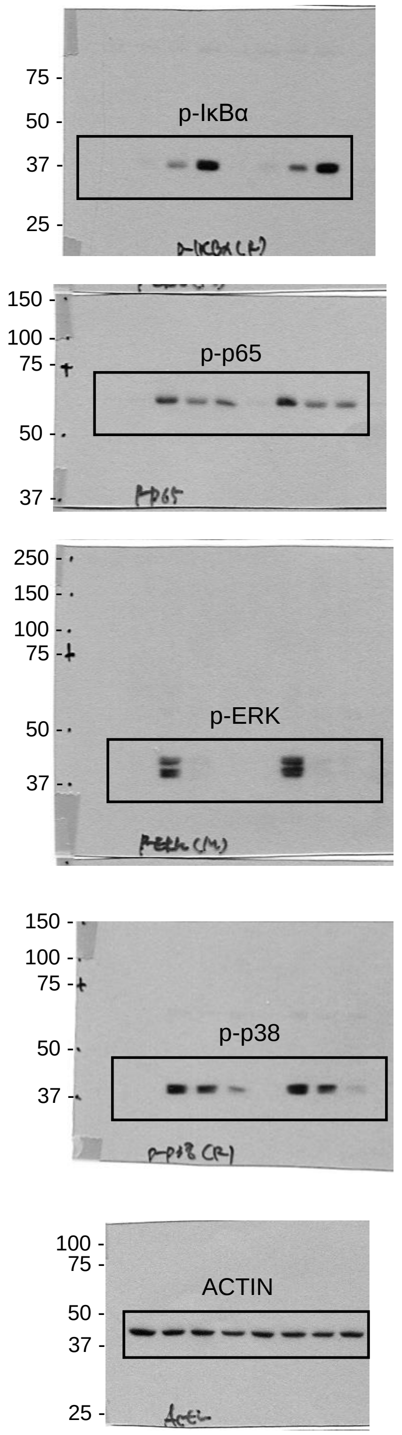

Supple Fig 4A

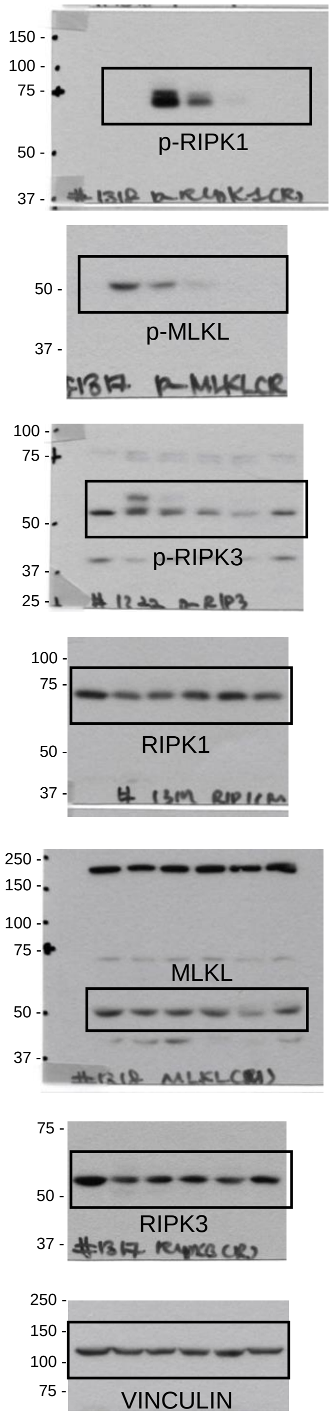

Supple Fig 4B

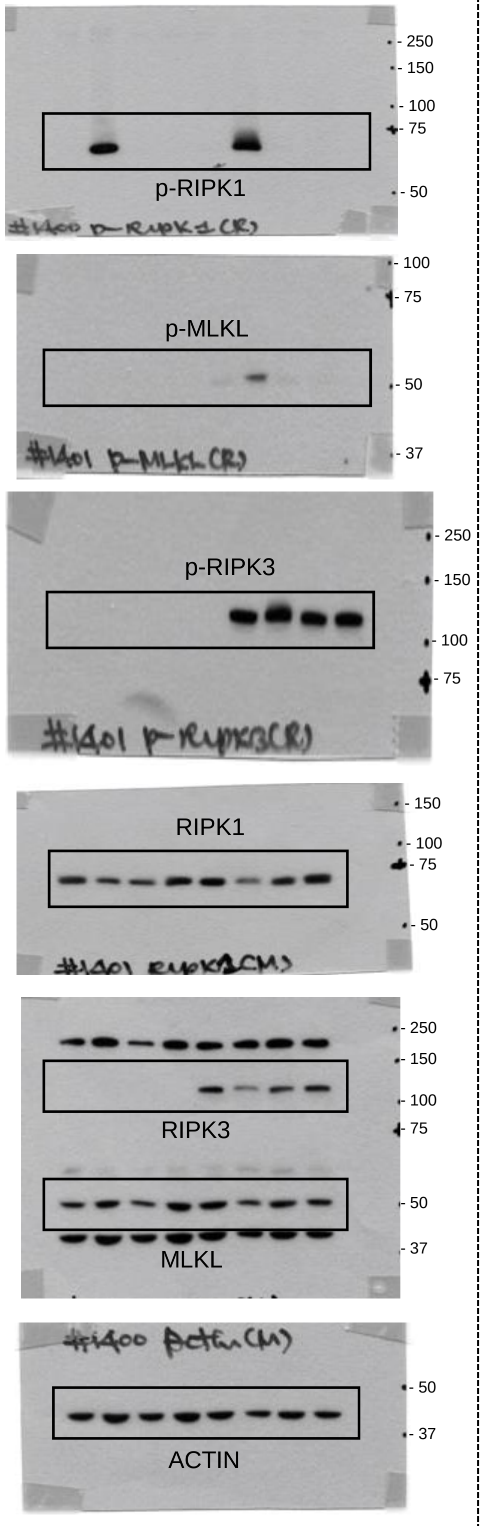

Supple Fig 4C

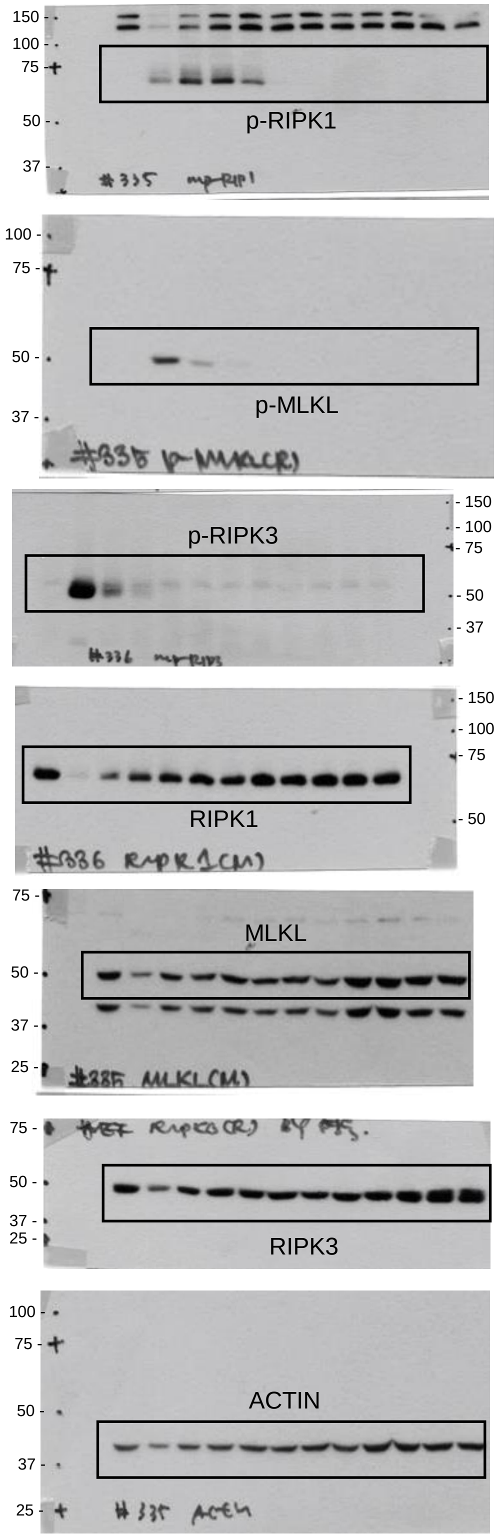

Supple Fig 4D (left)

Supple Fig 4D (right)

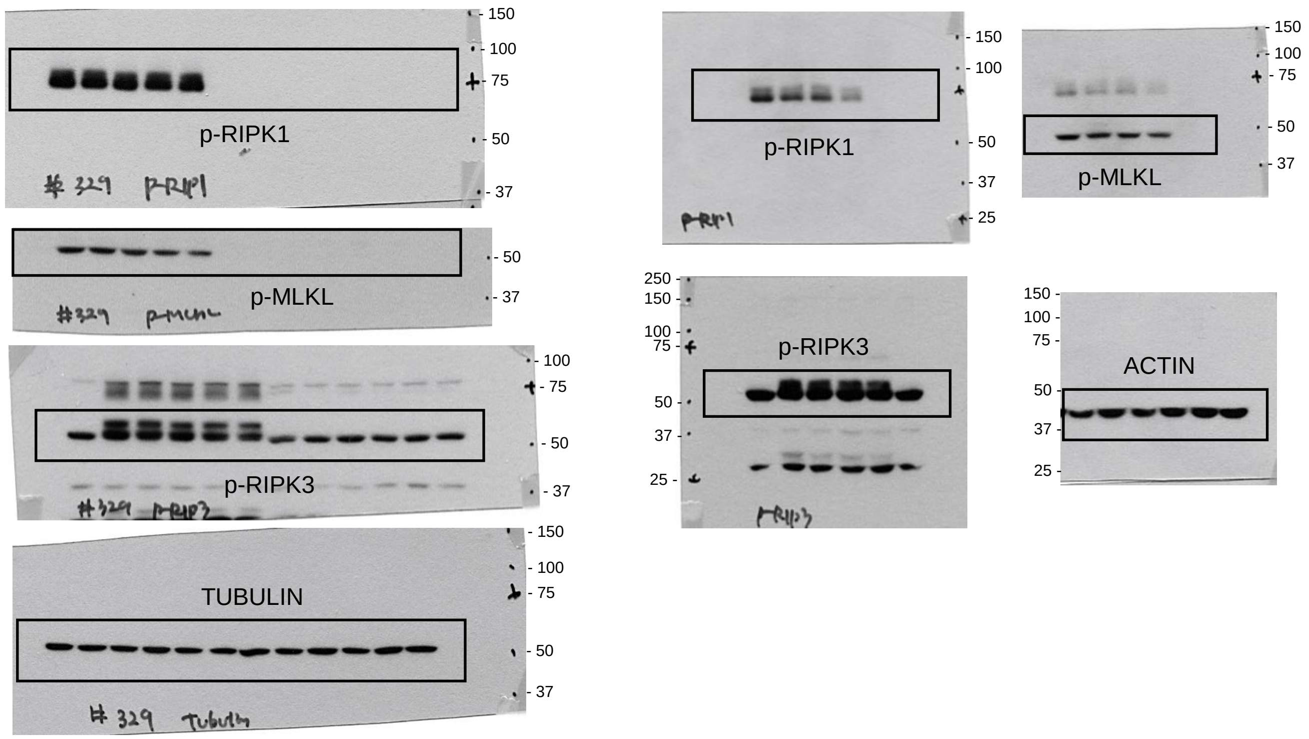

Supple Fig 4E

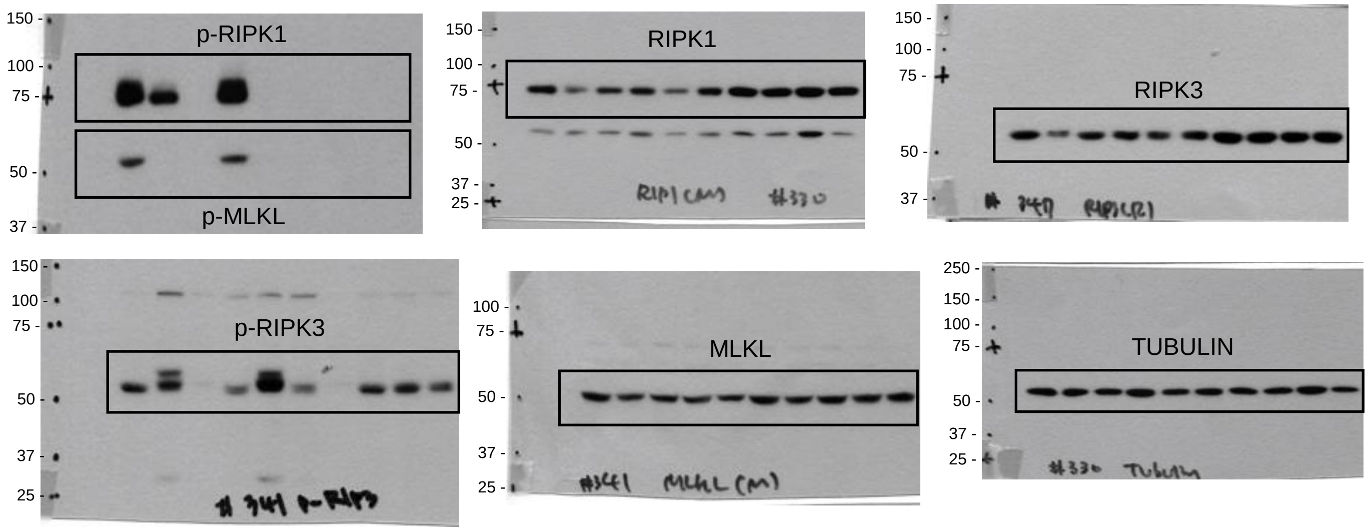

Supple Fig 4F

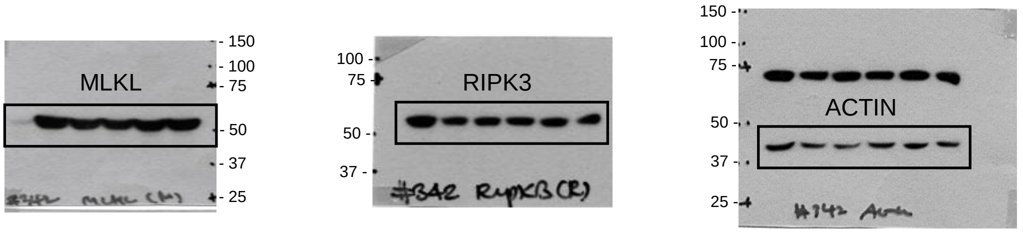

Supple Fig 4H

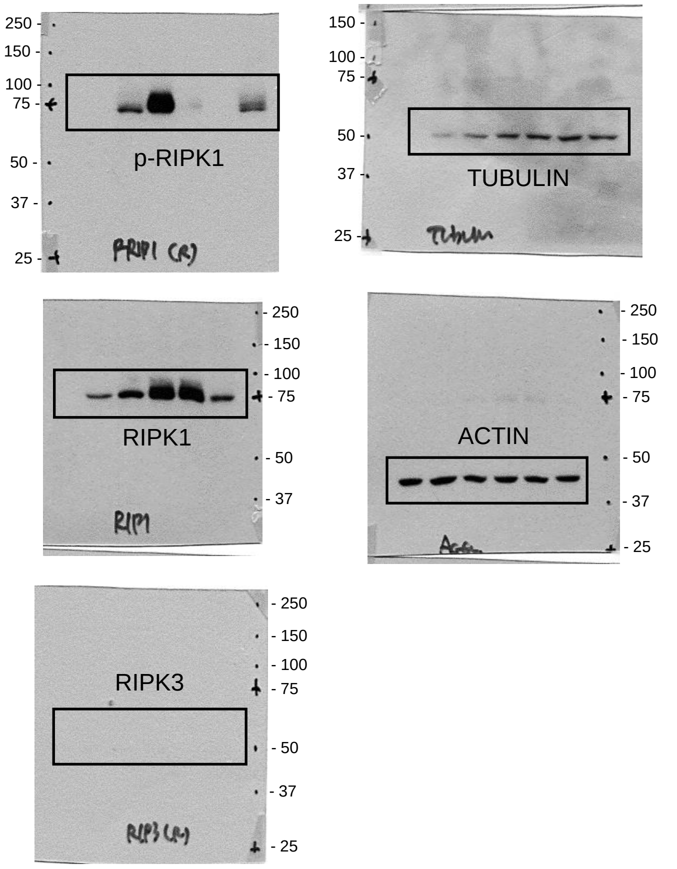

Supple Fig 4I

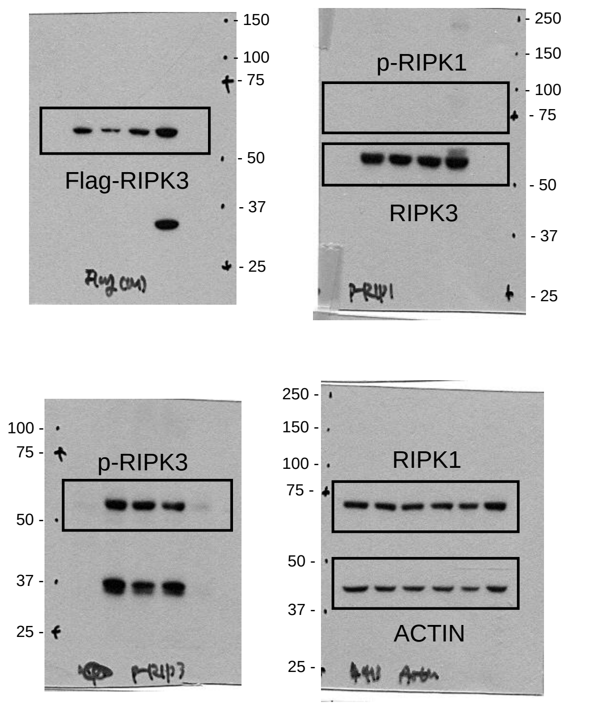

Supple Fig 4J (left)

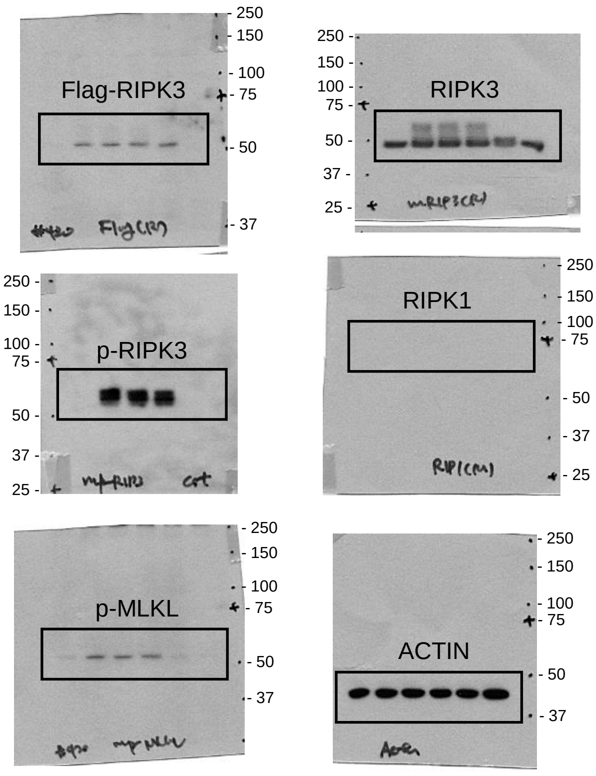

Supple Fig 4J (right)

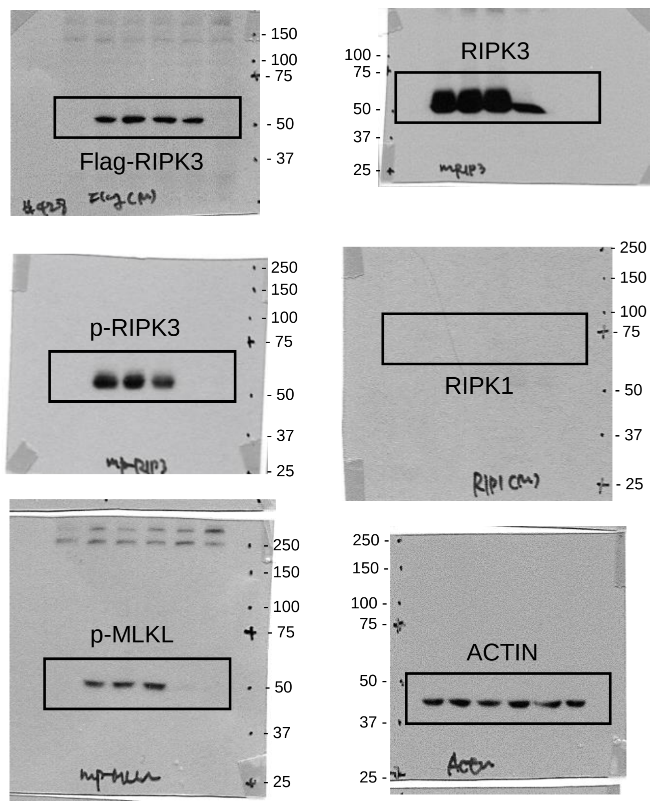

Supple Fig 5A (left)

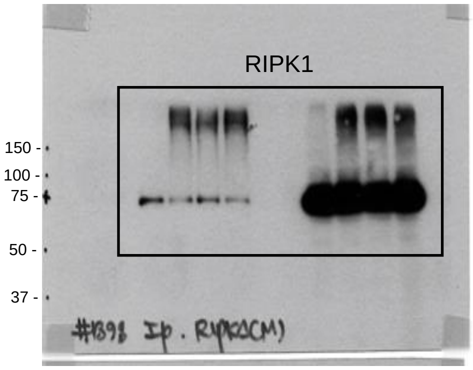

Supple Fig 5A (right)

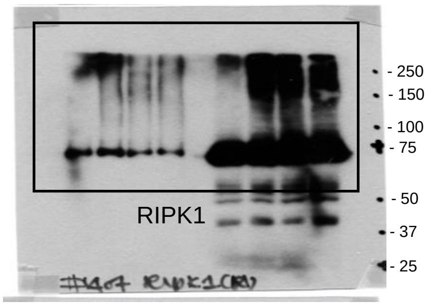

Supple Fig 5B

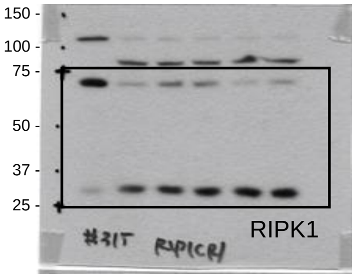

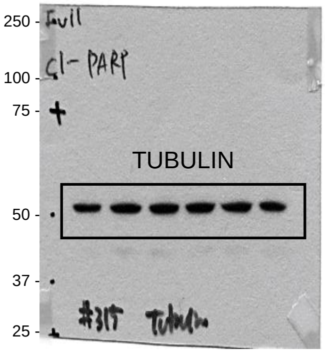

Supple Fig 6B

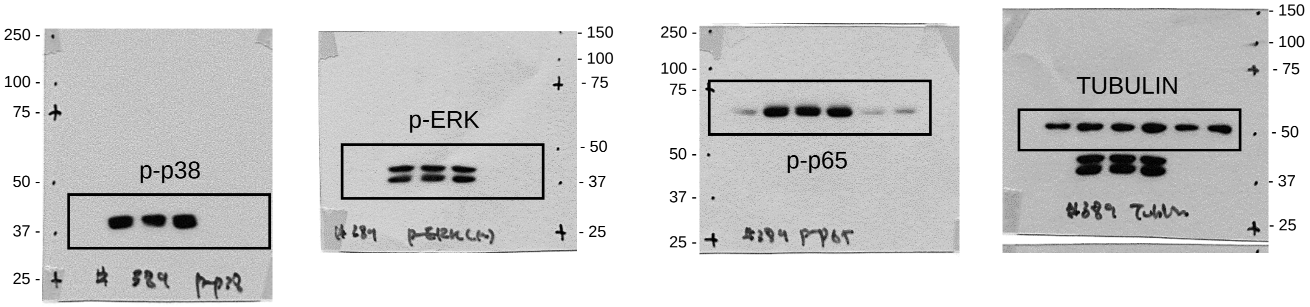

Supple Fig 6C

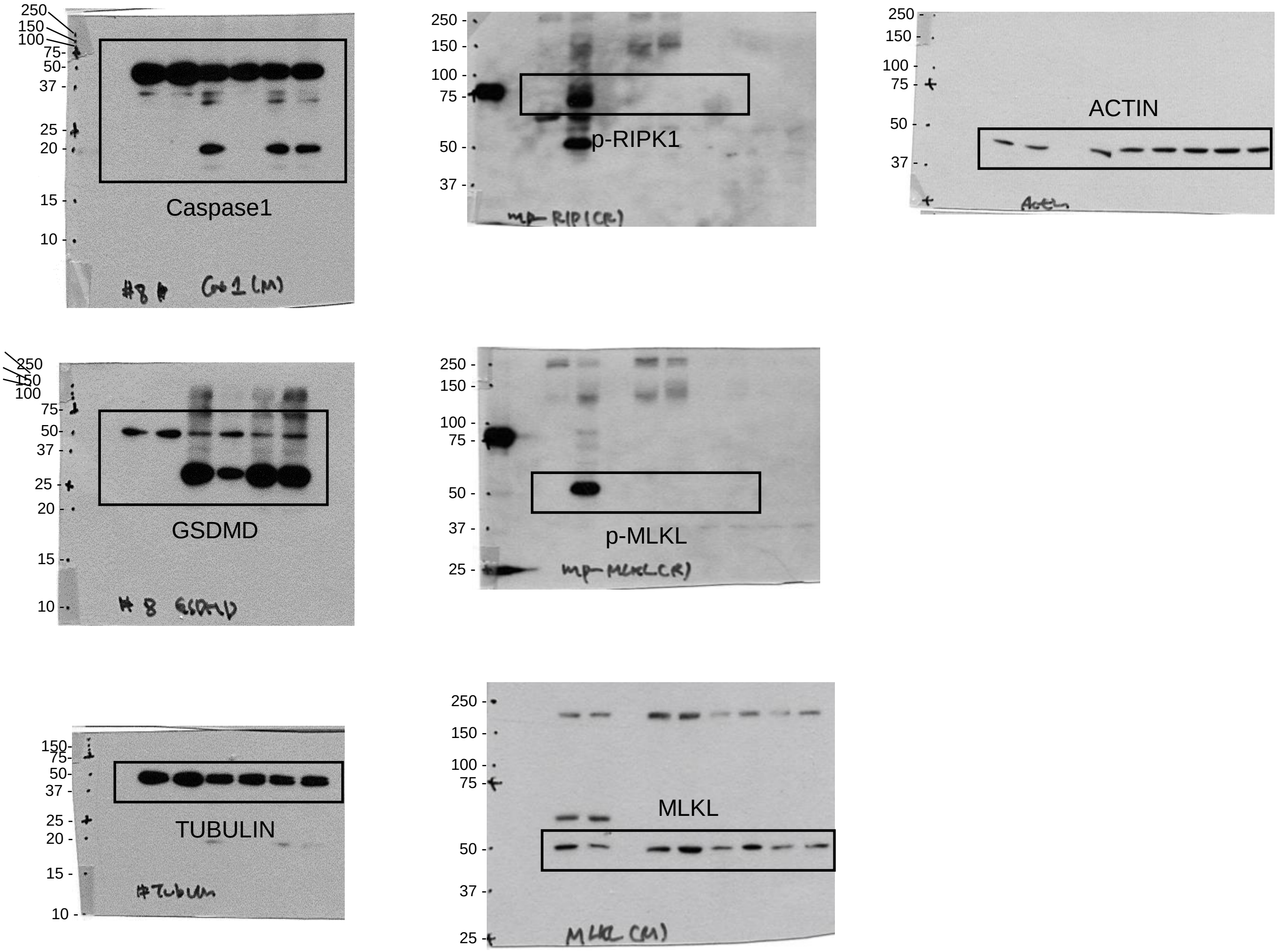

Supplement: Supplementary file 4 — Kim et al_uncropped Image [file 41419_2025_7754_MOESM4_ESM.pdf]
